# Bacterial diversity dynamics in microbial consortia selected for lignin utilization

**DOI:** 10.1371/journal.pone.0255083

**Published:** 2021-09-13

**Authors:** Isis Viana Mendes, Mariana Botelho Garcia, Ana Carolina Araújo Bitencourt, Renata Henrique Santana, Philippe de Castro Lins, Rafaella Silveira, Blake A. Simmons, John M. Gladden, Ricardo Henrique Kruger, Betania Ferraz Quirino

**Affiliations:** 1 Embrapa Agroenergia, Parque Estação Biológica (PqEB), PqEB s/n, Brasília, DF, Brazil; 2 Universidade de Brasília, Brasília, DF, Brazil; 3 Universidade Católica de Brasília, Brasília, DF, Brazil; 4 Instituto Federal de Brasília, Brasília, DF, Brazil; 5 Deconstruction Division, Joint BioEnergy Institute, Emeryville, California, United States of America; 6 Department of Biomass Science and Conversion Technology, Sandia National Laboratories, Livermore, California, United States of America; Friedrich Schiller University, GERMANY

## Abstract

Lignin is nature’s largest source of phenolic compounds. Its recalcitrance to enzymatic conversion is still a limiting step to increase the value of lignin. Although bacteria are able to degrade lignin in nature, most studies have focused on lignin degradation by fungi. To understand which bacteria are able to use lignin as the sole carbon source, natural selection over time was used to obtain enriched microbial consortia over a 12-week period. The source of microorganisms to establish these microbial consortia were commercial and backyard compost soils. Cultivation occurred at two different temperatures, 30°C and 37°C, in defined culture media containing either Kraft lignin or alkaline-extracted lignin as carbon source. iTag DNA sequencing of bacterial 16S rDNA gene was performed for each of the consortia at six timepoints (passages). The initial bacterial richness and diversity of backyard compost soil consortia was greater than that of commercial soil consortia, and both parameters decreased after the enrichment protocol, corroborating that selection was occurring. Bacterial consortia composition tended to stabilize from the fourth passage on. After the enrichment protocol, Firmicutes phylum bacteria were predominant when lignin extracted by alkaline method was used as a carbon source, whereas Proteobacteria were predominant when Kraft lignin was used. Bray-Curtis dissimilarity calculations at genus level, visualized using NMDS plots, showed that the type of lignin used as a carbon source contributed more to differentiate the bacterial consortia than the variable temperature. The main known bacterial genera selected to use lignin as a carbon source were *Altererythrobacter*, *Aminobacter*, *Bacillus*, *Burkholderia*, *Lysinibacillus*, *Microvirga*, *Mycobacterium*, *Ochrobactrum*, *Paenibacillus*, *Pseudomonas*, *Pseudoxanthomonas*, *Rhizobiales* and *Sphingobium*. These selected bacterial genera can be of particular interest for studying lignin degradation and utilization, as well as for lignin-related biotechnology applications.

## 1. Introduction

The world is looking for petroleum substitutes, both for energy and for making products that are clean and renewable [1–4]. Lignocellulosic biomass is already being successfully used industrially for these purposes, but its full potential has not been reached [5, 6]. Plant biomass has cellulose, hemicellulose and lignin as its main components. Lignin is the second most abundant biopolymer in nature, accounting for 5–35% of plant biomass composition [7, 8]. While there are a number of industrial applications for cellulose and hemicellulose that range from the production of the biofuel ethanol to the sugar substitute xylitol, applications for lignin are more restricted. In fact, it is quite common to burn lignin for energy, a low technology application that does not explore its potential as a natural source of phenolic compounds of industrial interest [9]. In a biorefinery model, lignin can be depolymerized and used to make value-added compounds, such as guaiacol, syringaldehyde, vanillin and ferulic acid [10]. These monomers can be used as the building blocks for bioproducts, phenolic resins, carbon fibers and nanomaterials [10–17].

Chemically, lignin is a phenolic heteropolymer formed by the dehydrogenation polymerization of p-hydroxyphenyl (H), guaiacil (G) and syringyl (S) units, derived from the monolignols coumaryl, coniferyl and synaphyl alcohol, respectively [18]. Lignin’s monomers are interconnected by ester, ether, C-C and β-O,4 bonds [18–20]. Coupling of these units by different bonds is what confers lignin its recalcitrance to enzymatic degradation. Despite lignin’s structural complexity, fungi and bacteria are capable of degrading it using the oxyreductase enzymes laccase, manganese peroxidase, versatile peroxidase and DyP peroxidase [20, 21]. Lignocellulosic biomass degradation by biological models, whether by microorganisms or enzymes alone, is of great interest as it occurs in mild conditions of temperature, pressure and pH [22]. Fungi from the Ascomycota and Basidiomycota phyla have been classified as lignin degrading [23]. Bacteria can also use lignin as carbon source, yet fewer ligninolytic bacterial species are known [24]. Studies show that when compared to fungi, bacteria ligninolytic enzymes can depolymerize lignin into a more restricted number of aromatic compounds, which would facilitate their use in biotechnological applications [9, 25].

Bacteria from the phyla Phylobacteria, Firmicutes and Bacterioidetes are capable of producing enzymes to breakdown lignin [24]. From a biotechnology perspective, bacterial diversity can be explored in search of new and better ligninolytic enzymes [26]. Previous studies have shown that ligninolytic bacterial species were successfully identified in soil-enriched microbial consortia for lignin degradation [10, 27–29]. Soil has a complex composition which includes microbial communities that perform multiple activities such as lignin degradation [27, 30]. However, the complexity of soil’s microbial communities can be overwhelming. A strategy to bypass this problem is to selectively cultivate specific microorganisms, such as those that can live using lignin as a sole carbon source. The process of enrichment of microbial consortia decreases functional complexity by converging the microbial community to similar biochemical functions facilitating identification of ligninolytic groups [10, 31].

In this work, an enrichment strategy was applied to establish microbial consortia selected to use lignin as carbon source. To this end, two sources of microorganisms (i.e., commercial soil and backyard compost soil), two cultivation temperatures (i.e., 30°C and 37°C) and two sources of lignin (i.e., alkaline-extracted and Kraft lignin) were used to establish eight microbial consortia in defined laboratory medium. Cultivation occurred over a period of 12 weeks, in which an aliquot of the consortia was inoculated into fresh medium every two weeks, totaling 6 subculturing cycles or passages. To understand the dynamics of microbial selection for the ability to utilize lignin as carbon source, we analyzed Illumina 16S rDNA gene sequence (iTag) phylogenetic profiling of bacteria data from these eight microbial consortia over six passages.

## 2. Materials and methods

### 2.1. Establishment of microbial consortia

**Fig 1:**
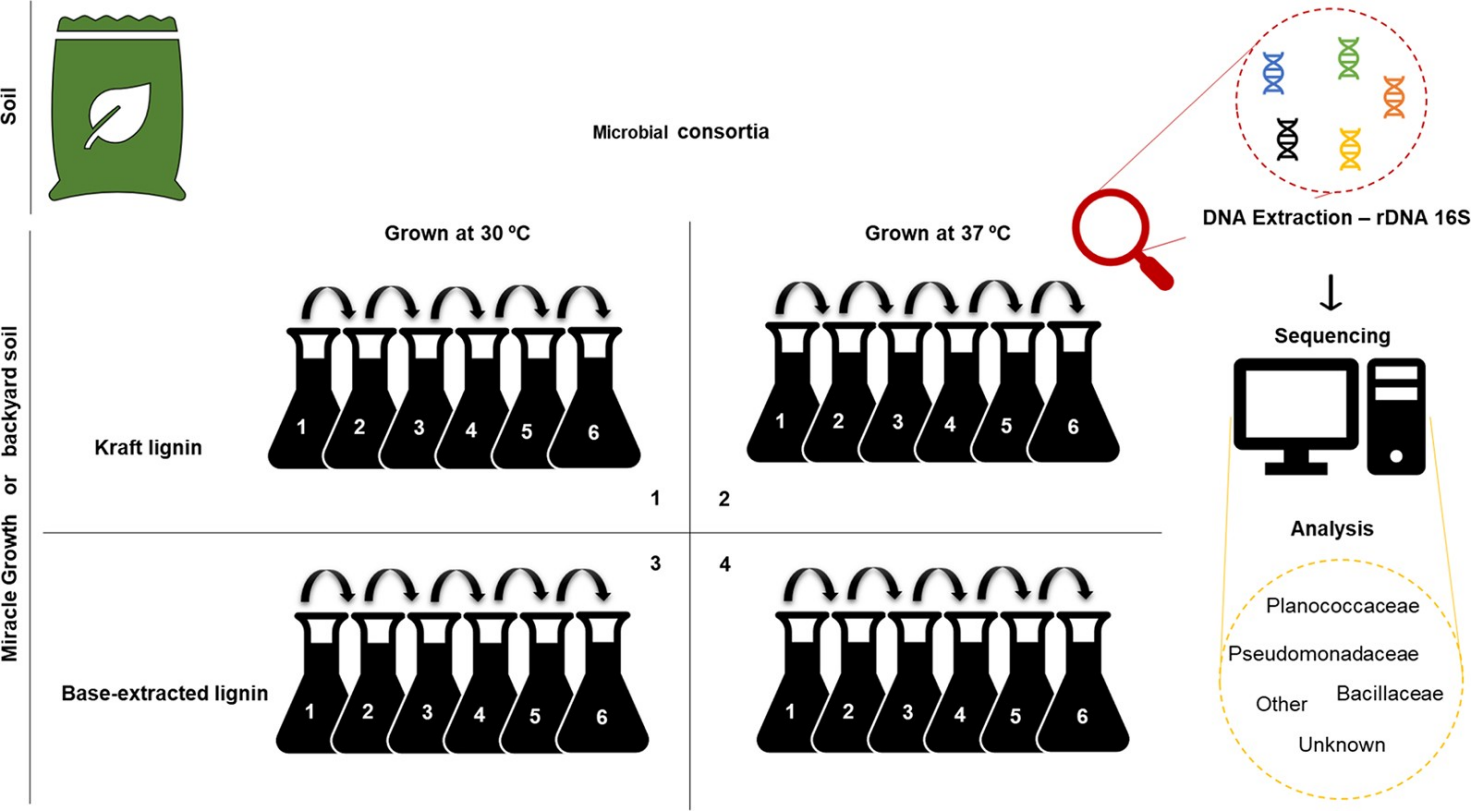
Schematic of enrichment experiment strategy. Eight microbial consortia enriched for microorganisms able to utilize lignin (i.e., base-extracted or Kraft) as carbon source. Four of the consortia had back yard soil and other four had MG soil as the as the original source of microorganisms. The enrichment experiments were conducted in aerobic conditions at 30°C and 37°C. Every two weeks, an aliquot of the growth medium containing microorganisms was transferred to fresh culture medium. The consortia were enriched over six cycles (passages), totaling 12-weeks. Samples were taken for each passage, DNA was extracted and the ribosomal gene 16S rDNA was amplified and sequenced.

For the establishment of microbial consortia, two sources of microorganisms were used: Miracle Growth commercial substrate and backyard compost soil which was obtained from Alameda County (California, USA). Commercial Kraft lignin and lignin from switchgrass extracted by alkaline method were used as carbon sources. The alkaline method for lignin extraction consisted in grinding 12.5 g of biomass and adding to it 250 mL of 0.5 M NaOH were added to 12.5 g of biomass and the solution was sterilized by autoclaving for 30 min at 121°C. Subsequently, H_2_SO_4_ 5 M was added until the pH of 7 was obtained. The solution was kept for 24 hours at 4°C, and then centrifuged at 10,000 x g for 30 min at 4°C. The supernatant was transferred to a second flask and centrifuged at 18,514 x g for 1 hour at 4°C; and the new supernatant was sterilized with 0.45 μm pore filter. A total of 50 mL of M9 minimal medium with trace elements [32] was added to eight 200 mL Erlenmeyer flasks (Fig 1). To each flask, 0.5 g of either Miracle Growth or backyard soil was inoculated. As carbon source, either 0.5 g/L of Kraft lignin (Sigma-Aldrich 471003) or 10% (w/w) of alkaline extract lignin was added. The cultures were kept at aerobic conditions with shaking at 200 rpm, either at 37°C or 30°C. For the enrichment process, a 2 mL aliquot of the microbial consortia was transferred to a new flask under the same conditions every two weeks, in a total of six passages [33]. At different cultivation times, 20 mL of each enriched culture were collected and then centrifuged at 20,000 g for 10 min at 4°C. The obtained microbial pellet was stored at -80°C until DNA extraction [33].

### 2.2. Illumina sequencing of 16S rDNA region

To identify the bacteria present in each consortium at each timepoint, DNA extraction was performed using All Prep DNA/RNA kit (QIagen) following the manufacturer’s instructions. DNA was then used as template in polymerase chain reactions (PCR) to amplify the copies of the 16S rDNA V4-V5 region. For this the FW (515F): 5’GTG CCA GCM GCC GCG GTAA 3’ and RV (805R): 5’ GGA CTA CHV GGG TWT CTA AT 3’ primers were used [34]. The fragments were analyzed using Bioanalyzer for quality control. Sequencing was according to JGI guidelines (Joint Genome Institute, CA, personal communication) [34] on the Miseq platform (Illumina) by pair end (2 x 150 bp) sequencing by the Joint Genome Institute (Walnut Creek, CA, USA) [34].

### 2.3. Bacterial diversity analyses

The 16S rDNA amplicons were grouped and classified into their respective operating taxonomic units (OTUs) using iTagger 2.0 program with the Usearch tool using 97% similarity by the Joint Genome institute (JGI) [35]. Silva SSU database was used to make taxonomic notes for 16S rRNA and Silva LSU for 18S rRNA [36, 37]. iTag data from cultures inoculated with Miracle Growth or Backyard soil were subjected to diversity analysis. Operational taxonomic units (OTUs) were analyzed using Quantitative Insights in Microbial Ecology (QIIME) software [38]. To calculate the alpha diversity of communities, the following scripts were employed: “alpha_rarefaction.py” with a sample of 150,000 bacterial OTUs. For richness and diversity analysis, the following metrics were calculated: observed OTUs, Chao1, Shannon, 1-Simpson, Good’s coverage and PD whole tree. To calculate the absolute abundance of each community, the script “summarize_taxa.py” was used. In Excel (Microsoft Office), the absolute abundance data for each community was calculated. For the consortia inoculated with Miracle Growth soil, only the main bacterial OTUs with relative abundance above 1.0% were used to generate taxonomy graphs. For the consortia established from backyard compost soil, bacterial OTUs with relative abundance above 1.0% were used to generate taxonomy graphs. The relative abundance data was used to create bar graphs with the main bacterial families present in the consortia. To analyze consortia dissimilarity, genus data from different passages was used in Bray-Curtis based non-metric multidimensional scaling (NMDS) [39]. Bray Curtis matrix PERMANOVA testing using the adonis function of vegan package in R [39]. Additionally, vectors of taxa driving clusters were found using the envfit function of vegan package in R performing 10,000 permutations [39]. The data of the most abundant genera with relative abundance above 1.0% that were present in the sixth passage of each microbial consortium were used for construction of a Venn diagram [40].

## 3. Results

### 3.1. Alpha diversity of microbial consortia

As shown in Fig 1, eight microbial consortia enriched for the ability to utilize lignin as a carbon source were obtained. Four of the consortia were obtained using Miracle growth (MG) commercial soil as a source of microorganisms, and four others used backyard (BY) compost soil. Two types of carbon source were used, base-extracted lignin (BE Lig) and Kraft lignin. Growth under aerobic conditions occurred at 30°C and 37°C.

16S rDNA sequencing data from the original soil (control) were used to estimate the diversity present in each consortium prior to the enrichment process for lignin utilization. As shown on Table 1, Good’s coverage of 99% and 100% for all passages of all consortia indicate that the number of sequences sampled was sufficient to represent the diversity of the consortia studied (Table 1 and S1 and S2 Tables). The original soil sample, MG or BY, shows a higher number of bacterial operating taxonomic units (OTUs) than that found for the sixth passage for both types of lignin substrate and for both temperatures. Chao 1 index shows that richness decreased both for MG and BY derived consortia at both temperatures, when one compares numbers for the bacterial consortia found in the original soil samples to those found in the sixth passage. Diversity indexes (i.e., PD whole tree, 1-Simpson and Shannon) show a decrease in diversity in the bacterial consortia present in the sixth passage compared to the original ones, both for MG and BY derived consortia and for both temperatures. It is also noticeable that the indexes obtained show that bacterial richness and diversity is greater in backyard-derived samples in comparison to MG-derived samples.

**Table 1:** Diversity indexes for the original substrate (i.e., MG and BY compost soils) and at the sixth passage of enriched bacterial consortia, for base-extracted and Kraft lignin utilization cultivated at 30°C and 37°C.

Sample	Sample	OTU	Goods’	Chao1	PD wholetree	Shannon	1-Simpson
			coverage				
	MG_BE_30°C	307	100%	346.723	32.327	4.091	0.861
	MG_BE_37°C	308	100%	340.453	32.375	4.093	0.862
	MG_Kraft_30°C	309	100%	346.310	32.568	4.094	0.862
**Original**	MG_Kraft_37°C	309	100%	349.022	32.390	4.093	0.862
**Soil**	BY_BE_30°C	1,061	99%	1,175.026	72.810	7.991	0.990
	BY_BE_37°C	1,061	99%	1,186.272	72.873	7.991	0.990
	BY_Kraft_30°C	1,061	99%	1,173.724	72.804	7.993	0.990
	BY_Kraft_37°C	1,059	100%	1,172.735	72.871	7.992	0.990
	MG_BE_30°C	209	100%	227.927	22.425	3.467	0.803
	MG_BE_37°C	171	100%	158.112	12.750	3.428	0.766
	MG_Kraft_30°C	144	100%	184.670	18.653	2.662	0.682
**6** ^**th**^ **Passage**	MG_Kraft_37°C	107	100%	114.618	10.081	4.104	0.904
	BY_BE_30°C	230	100%	248.303	22.722	4.075	0.885
	BY_BE_37°C	185	99%	185.470	15.421	4.930	0.934
	BY_Kraft_30°C	171	100%	173.545	18.517	3.770	0.839
	BY_Kraft_37°C	101	100%	109.451	13.352	3.500	0.806

### 3.2. Dynamics of taxonomic profiles during enrichment

**Fig 2:**
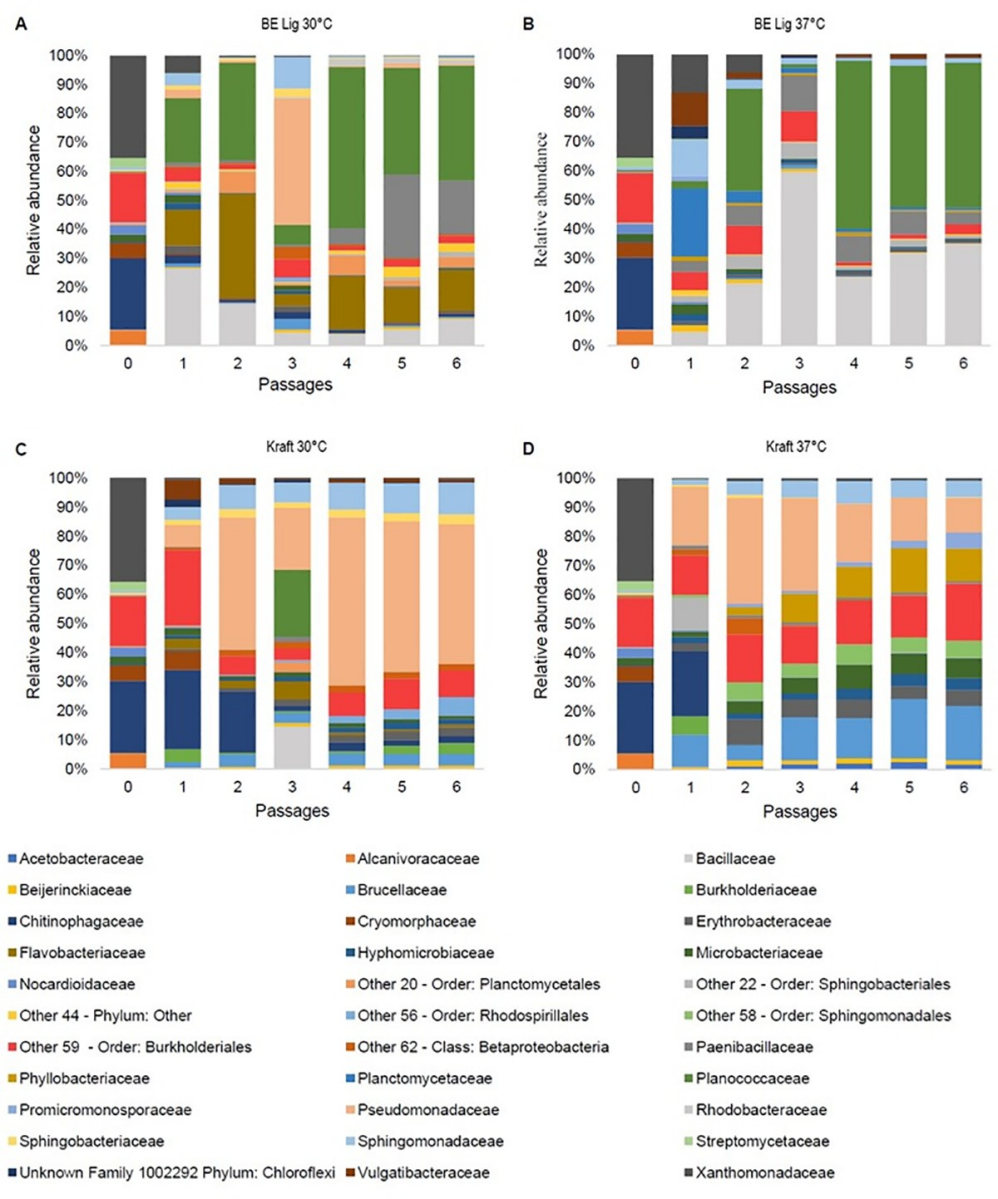
Family level taxonomic profile of bacteria at present in consortia obtained from MG soil over successive passages in enrichment experiment. M9 medium containing either base-extracted lignin, BE Lig, (panels A and B) or Kraft lignin (panels C and D) was initially inoculated with MG soil (MG) as the source of microorganisms. Experiments were performed at two temperatures. 30°C (panels A and C) and 37°C (panels B and D). Every 2 weeks an aliquot was transferred to fresh growth medium for enrichment in successive passages (0, 1, 2, 3, 4, 5 and 6). Bacterial families are depicted by different colors. For the families classified as Other and Unknown, we were unable to obtain the taxonomic affiliation at the family level, so the closest previous hierarchical level is provided.

**Fig 3:**
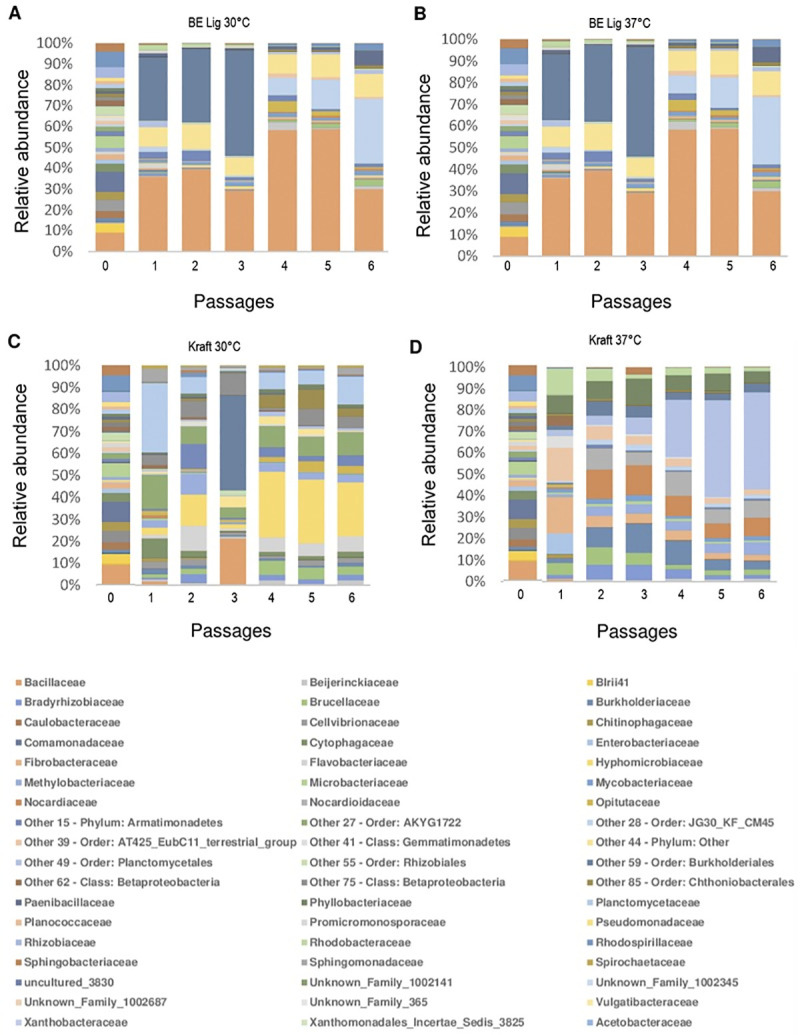
Family level taxonomic profile of bacteria at present in consortia obtained from backyard (BY) soil over successive passages in enrichment experiment. M9 medium containing either base-extracted lignin, BE Lig, (panels A and B) or Kraft lignin (panels C and D) was initially inoculated with backyard (BY) as the source of microorganisms. Experiments were performed at two temperatures. 30°C (panels A and C) and 37°C (panels B and D). Every 2 weeks an aliquot was transferred to fresh growth medium for enrichment in successive passages (0, 1, 2, 3, 4, 5 and 6). Bacterial families are depicted by different colors. For the families classified as Other and Unknown, we were unable to obtain the taxonomic affiliation at the family level, so the closest previous hierarchical level is provided.

To understand the dynamics during the enrichment process, taxonomic affiliation at family level of 16S rDNA amplicons to operational taxonomic units (OTUS) was performed using 97% sequence similarity [34]. Figs 2 and 3 show family-level taxonomy data of bacterial consortia over the six passages.

The main bacterial families with relative abundance above 1% in any of the passages were represented in taxonomy graphs (Figs 2 and 3 and S4 and S5 Tables). At the end of the enrichment process on the sixth passage, the families present in the microbial consortium derived from MG soil enriched with lignin extracted by alkaline method and grown at 30°C (i.e., BE-Lig at 30° C) represent more than 95% of the abundance total relative abundance, the most abundant families present being: Planococcaceae (37.8%), Paenibacillaceae (17.6%), Flavobacteriaceae (13.4%), Bacillaceae (8.6%), Other 20 (Order: Planctomycetales) (2.9%), Other 22 (Order: Sphingobacteriales) (3.5%) and Other 59 (Order: Burkholderiales) (2.4%) (Fig 2A). For the BE-Lig consortium at 37° C (i.e., MG BE-Lig at 37° C), the families present in the sixth passage represent more than 93% of the total relative abundance, with the most abundant families being: Planococcaceae (46.59%), Bacillaceae (32.65%), Paenibacillaceae (3.89%), Other 59 (Order: Burkholderiales) (3.2%), and Sphingomonadaceae (1.51%) (Fig 2B). For consortia derived from Miracle Growth soil, enriched with Kraft lignin and grown at 30° C (i.e. Kraft at 30°C), the families present in the sixth passage represent more than 96% of the total relative abundance, the main ones are: Pseudomonadaceae (46.35%), Sphingomonadaceae (10.60%), Other 59 (Order: Burkholderiales) (8.84%), Other 56 (Order: Rhodospirillales) (6.22%), Brucellaceae (3.93%), Erythrobacteraceae (2.87%), Burkholderiaceae (3.59%) and Chitinophagaceae (2.34%) (Fig 2C). For consortia derived from Miracle Growth soil, enriched with Kraft lignin and grown at 37°C (i.e. Kraft at 37°C) the families present in the sixth passage represent more than 92% of the total relative abundance, the main ones being: Other 59—Order: Burkholderiales (18, 0224%), Brucellaceae (17.3061%), Pseudomonadaceae (11.0626%), Phyllobacteriaceae (10.3328%), Microbacteriaceae (6.3061%), Other 58—Order: Sphingomonadales (5.3817%), Sphingomonadaceae (5.1807%), Promicromonosporaceae (5.0468%), Erythrobacteraceae and (4.9365%) and Hyphomicrobiaceae (3.8272%) (Fig 2D).

A similar analysis was performed for backyard compost derived consortia (Fig 3 and S5 Table). At the end of the enrichment process at the sixth passage, bacterial families that showed relative abundance greater than 1% accounted for 81%, 81%, 84% and 87% of the total abundance present in the consortia that used base-extracted lignin at 30°C and at 37°C (i.e., BE-Lig at 30°C, BE-Lig at 37°C) and those that used Kraft lignin at 30°C and at 37°C (i.e., Kraft at 30°C, and Kraft at 37°C), respectively. Predominant bacterial families at the sixth passage for consortia that used base-extracted lignin as carbon source at temperatures 30°C and 37°C were the same and were: Other 59 (Order: Burkholderiales) (29.651%), Bacillaceae (28.628%), Paenibacillaceae (10.402%), Sphingomonadaceae (6.908%), Xanthobacteraceae (2.944%) and Brucellaceae (2.837%). For backyard compost derived consortia that used Kraft lignin as carbon source cultivated at 30°C, the predominant bacterial families at the sixth passage were: Methylobacteriaceae (22.516%), Sphingomonadaceae (11.565%), Other 59 (Order: Burkholderiales) (9.760%), Hyphomicrobiaceae (6.466%), Pseudomonadaceae (4.486%), Other 55 (Order: Rhizobiales) (4.357%), Rhizobiaceae (3.560%), Burkholderiaceae (3.434%), Other 49 (Order: Planctomycetales) (3.210%), Xanthomonadaceae (2.924%), Microbacteriaceae (2.919%), Erythrobacteraceae (2.421%), Bradyrhizobiaceae (2.341%), Chitinophagaceae (2.176%) and Beijerinckiaceae (2.041%). Finally, for backyard compost derived consortia that used Kraft lignin as carbon source cultivated at 37°C, the predominant bacterial families at the sixth passage were: Phyllobacteriaceae (44.365%), Mycobacteriaceae (8.472%), Nocardiaceae (7.829%), Hyphomicrobiaceae (5.818%), Sphingomonadaceae (4.966%), Promicromonosporaceae (4.291%), Burkholderiaceae (3.903%), Erythrobacteraceae (2.673%), Brucellaceae (2.540%), Other 59 (Order: Burkholderiales) (2.314%).

**Fig 4:**
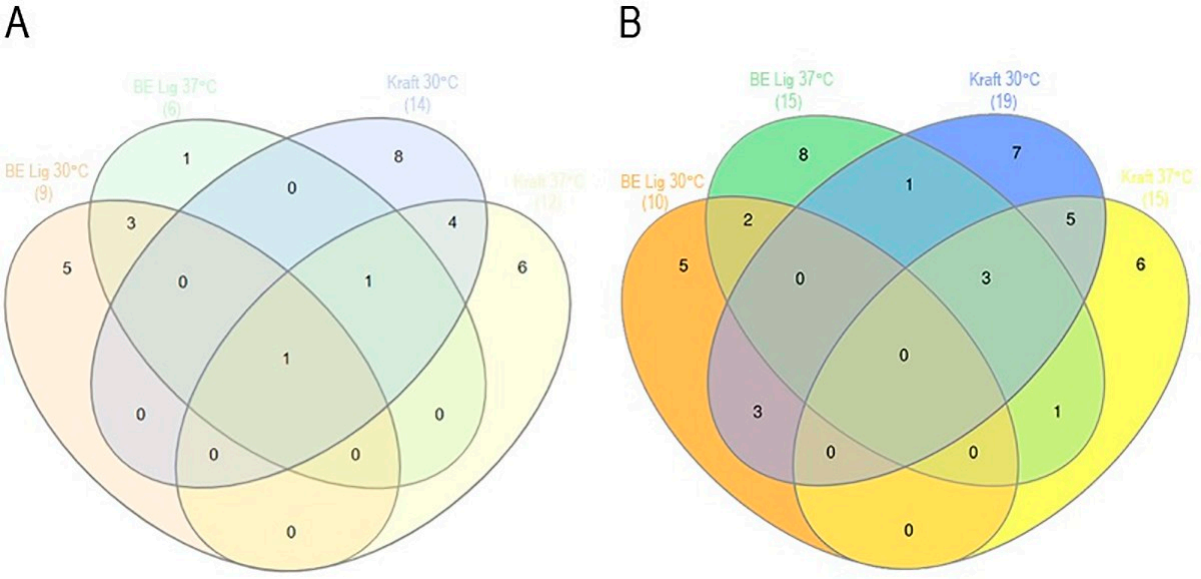
Venn diagram of bacterial genera present at the 6^th^ passage of enrichment in consortia obtained using either base-extracted (BE-Lig) or Kraft lignin (Kraft) as carbon source at cultivation temperatures of 30°C and 37°C. Shown in parenthesis is the number of genera for each growth condition with relative abundance above 1%. (A) Bacterial consortia derived from MG compost. (B) Bacterial consortia derived from backyard (BY) compost.

The Venn diagram in Fig 4A (also see S1 Data) was constructed from genera in the sixth passage with relative abundance above 1%. It shows the 41 most abundant bacterial genera (i.e., from 498 MG-derived consortia present at the sixth passage). A total of 9 genera were present in the consortium that used base-extracted lignin as carbon source at 30°C (BE-Lig 30°C); 6 genera were present in the consortium that used base-extracted lignin as carbon source at 37°C (BE-Lig 37°C); 14 genera were present in the consortium that used Kraft lignin as carbon source at 30°C (Kraft 30°C), and 12 genera were present in the consortium that used Kraft lignin as carbon source at 37°C (Kraft 37°C). Only one genus is shared by the four consortia, *Other* 120, an unknown genus of the Burkholderiales order. The consortia that used base-extracted lignin as carbon source (i.e., BE-Lig 30°C and BE-Lig 37°C) share three genera: *Bacillus*, *Lysinibacillus* and *Paenibacillus;* meanwhile, the consortia that used Kraft lignin (i.e., MG Kraft 30°C and MG Kraft 37°C) share four genera: *Altererythrobacter*, *Camelimonas*, *Ochrobactrum* and *Pseudomonas*. The genera *Sphingobium* was shared by the three consortia BE 37°C, Kraft 30°C and 37°C. The genera present exclusively at BE 30°C were Other 38 (Family: Flavobacteriaceae), Other 44 (Order: Sphingobacteriales), Other 86 (Phylum: Other), Other 92 (Order: Planctomycetales) and Other 109 (Family: Rhodobacteraceae). The genera present exclusively at BE Lig 37°C were: Microvirga. The genera that appeared exclusively in Kraft 30°C were: Burkholderia, Devosia, Other 45 (Family: Sphingobacteriaceae), Other 100 (Class: Alphaproteobacteria), Other 111 (Ordes: Rhodospirillales), Other 124 (Class: Betaproteobacteria), Sphingopyxis, and Taibaiella. The genera that appeared exclusively in Kraft 37°C were: Aminobacter, Inquilinus, Isoptericola, Microbacterium, Roseomonas and Uncultured_2490 (Family: Hyphomicrobiaceae).

The Venn diagram in Fig 4B (also see S1 Data) shows the 59 most abundant bacterial genera (i.e., from a total of 427) BY derived consortia present at the sixth passage. The diagram was constructed from genera in the sixth passage with relative abundance above 1%. A total of 10 genera were present in the consortium that used base-extracted lignin as carbon source at 30°C (BE 30°C); 15 genera were present in the consortium that used base-extracted lignin as carbon source at 37°C (BE 37°C); 19 genera were present in the consortium that used Kraft lignin as carbon source at 30°C (Kraft 30°C), and 15 genera were present in the consortium that used Kraft lignin as carbon source at 37°C (Kraft 37°C). The genera present exclusively at BE 30°C were *Lysinibacillus*, Other 42 (Family: Chitinophagaceae), Other 120 (Order: Burkholderiales), Unknown_Genus_1000421 (Class: Thermomicrobia), Unknown_Genus_1001041 (Order: Chthoniobacterales). The genera present exclusively at BE Lig 37°C were: *Brevibacillus*, *Mesorhizobium*, Other 52 (Class: Thermomicrobia), Other 53 (Class: Thermomicrobia), Other 86 (Phylum: Other), *Pseudoxanthomonas*, *Sphingopyxis*, Unknown_Genus_1000489 (Family: Paenibacillaceae). The genera that appeared exclusively in Kraft 30°C were *Azospirillum*, *Burkholderia*, *Dokdonella*, *Kaistia*, *Microvirga*, *Terrimonas*, uncultured_2499 (Family: Methylobacteriaceae). The genera that appeared exclusively in Kraft 37°C were *Hyphomicrobium*, *Isoptericola*, *Mycobacterium*, Other 106 (Family: Phyllobacteriaceae), Other 118 (Order: Burkholderiales), and *Rhodococcus*. There was no genus present in common for the four conditions (i.e., BE Lig 30°C, BE Lig 37°C, Kraft 30°C and Kraft 37°C). Only 2 genera were present when base-extracted lignin was used as carbon source at both cultivation temperatures (*Bacillus* and *Paenibacillus*). On the other hand, 8 genera were present when Kraft lignin was used as carbon source at both cultivation temperatures (*Afipia*, *Altererythrobacter*, *Aminobacter*, *Microbacterium* and Other 105 (Order: Rhizobiales).

### 3.3. Influence of temperature and type of lignin in the selection of bacteria

To evaluate how temperature and type of lignin (i.e., base-extracted or Kraft lignin) influenced the composition of microbial consortia during the enrichment process, genus level data were used for Bray-Curtis Non-Metric Multidimensional Scaling (NMDS) matrix dissimilarity analysis (Fig 4). NMDS graphs present coordinates that represent adjusted distances, therefore, it is possible to observe the dissimilarity between the taxonomic composition of samples.

**Fig 5:**
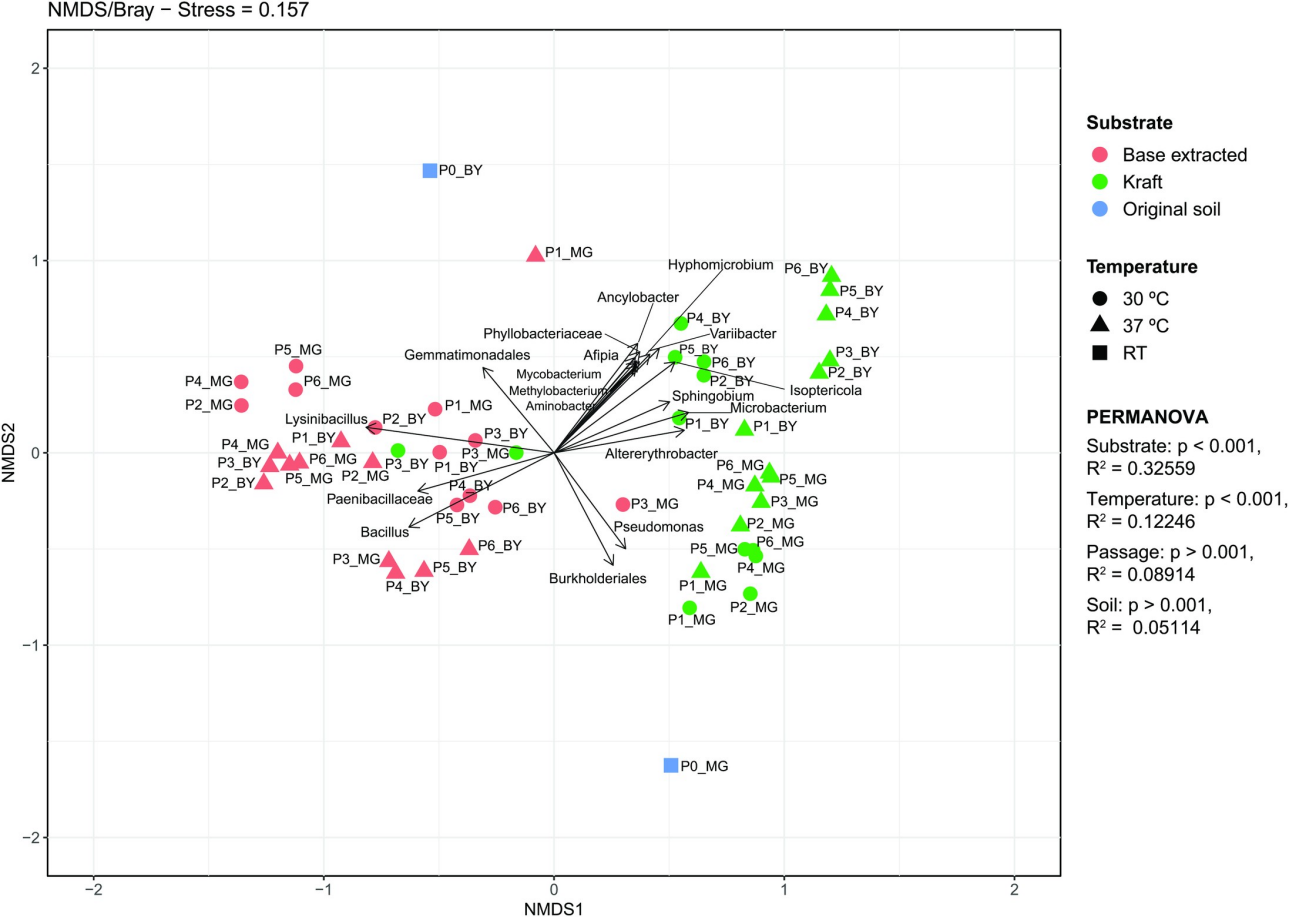
Bray-Curtis analysis of dissimilarity in the non-metric multidimensional scaling matrix (NMDS) to consortia composition at genus level in relation to the variables type of lignin used as carbon source and temperature. MG- and backyard (BY) compost derived consortia were analyzed. P0 refers to the original compost; “P1” to “P6” refer to the six passages (selection cycles); The stress number is given at the top. * Stress <0.05 excellent, <0.1 great, <0.2 good and<0.3 bad.

Fig 5 shows a stress number of 0.157 for the NMDS grouping of MG- and backyard (BY) compost-derived consortia, indicating that the representation (i.e., NMDS ordination) of the data is a good fit. Bacterial consortia present in the original composts, P0_MG and P0_BY, are highly dissimilar to each other, P0_MG being located in the lower right region of the graph, and P0_BY in the upper left region of the graph. Furthermore, these consortia are separated from all other ones shown in the graph. It is also interesting that consortia are separated in the graph according to the type of carbon source used during the selection process. Regarding the variable temperature, a pattern of dissimilarity between the samples cannot be observed, indicating that this variable does not appear to influence the composition of the consortia as strongly as with the type of lignin available as carbon source. To determine the statistical significance of this, PERMANOVA testing was used for the individually analyzing variables (Soil/MG and BY, Passage/0 to 6, Substrate/base extracted and Kraft lignin, Temperature/30°C and 37°C). As shown in the S1 File and Fig 5, the variable that best explained variation (~ 32%) of distances was Substrate (i.e., lignin type: base-extracted or Kraft lignin) for which an R^2^ of 0.32559 was obtained (p-value = 9.999e-05). Furthermore, temperature, passage and soil presented R^2^ values of 0.12246, 0.08914 and 0.05114, respectively. Fig 5 also shows vectors for the most statistically significant taxa driving the red and green clusters (based extracted lignin and Kraft lignin, respectively). At P ≤ 0.0001, taxa driving the red cluster were the genera *Bacillus* and *Lysinibacillus*, the family Paenibacillaceae, and order Gemmatimonadales, (see S2 File). Similarly, at P ≤ 0.0001, the taxa driving the green cluster are the genera *Pseudomonas*, *Afipia*, *Mycobacterium*, *Methylobacterium*, *Ancylobacter*, *Aminobacter*, *Hyphomicrobium*, *Variibacter*, *Sphingobium*, *Isoptericola*, *Altererythrobacter and Microbacterium; the* order Burkholderiales, and the family Phyllobacteriaceae (see S2 File).

## 4. Discussion

### 4.1. Consortia alpha diversity

Lignin is highly resistant to chemical as well as biological degradation [41]. However, some groups of fungi and bacteria have the functional machinery for lignin degradation. Although few bacterial ligninolytic enzymes are known, bacteria are an attractive option for studying because they can be grown to large scale and be genetically engineered easily [24]. In addition, prokaryotes are the most abundant group when it comes to microbial soil communities [42–44].

In this study, two sources of microorganisms (i.e., commercial MG soil and backyard compost soil), two different temperatures (i.e., 30°C and 37°C) and two types of lignin (i.e., base-extracted and Kraft) were used to maximize the different kinds of bacteria selected during enrichment. At the end of the protocol, eight microbial consortia enriched for the ability to degrade lignin were obtained. The use of enriched microbial consortia has been considered as a promising approach to identify microorganisms involved in lignin degradation and conversion [10, 27, 30]. The enrichment process is expected to select for microorganisms that carry enzymatic machinery to degrade lignin and use it as carbon source. In our study, there was an approximate 6- and 2- fold reduction in the number of OTUs present in the original soil samples and at the sixth passage of enrichment of MG derived and backyard compost derived consortia, respectively. Comparative analysis of richness and diversity indexes between the different consortia passages (i.e., number of OTUs observed, Chao 1, PD whole tree, Shannon and 1-Simpson) showed that there is an overall pattern of decreasing richness and diversity, similar to what was described by Yu et al. (2015) [27]. Nevertheless, there were small fluctuations in the values found for expected richness and diversity indexes when microbial consortia of successive passages are analyzed. It is important to point out that these consortia are composed of living microorganisms that interact in an intraspecific and interspecific manner; thus, the dynamics of the consortia tend to be constantly changing.

Similar results of decreased diversity were also observed by Moraes et al. (2018) [10]. when using an enrichment approach to obtain a microbial consortium (LigMet) from sugarcane plantation soil, using lignin as carbon source for 50 weeks. This decrease in diversity is an expected response as in each subculturing cycle there is a progressive selection of microorganisms that display adaptive characteristics. During the enrichment process, the different lignin substrates, temperatures and cultivation time used favor selection of families that have the enzymatic machinery necessary to degrade lignin, and resist toxicity of the compounds generated by this process, therefore some bacteria become dominant at the expense of others [10, 30].

Consortia started from backyard compost soil showed higher initial richness and diversity in comparison to consortia derived from MG soil, a commercial product. Furthermore, there is a legacy effect for the initial inoculum as the higher bacterial richness and diversity for backyard compost derived consortia persisted at the sixth passage after successive cycles of selection. Therefore, in future similar experiments if the intention is to maximize different taxa at the end of the selection process, it may be important to choose a highly diverse source of microorganisms for the initial inoculum.

Our data also show influence of the type of carbon source on richness and diversity indexes. At the end of the enrichment process, consortia that used lignin extracted by alkaline method as carbon source had a higher number of observed OTUs, a higher PD whole tree index and a higher Chao 1 richness index, when compared to the sixth passage of the consortia that used Kraft lignin. It is likely that the lignin extracted by the alkaline method from plant biomass presented some contamination with carbohydrates (i.e., hemicellulose), which would act as an alternative carbon source. This alternative carbon source may have allowed the presence of bacterial species that do not use lignin in the environment as an exclusive carbon source, and thus may have contributed to a higher richness and diversity.

### 4.2. Dynamics of taxonomic profiles during enrichment

In this study, sequencing data were analyzed down to the genus level, as it was not possible to perform taxonomic affiliation at the level of species. Analysis of the consortia bacterial composition based on the amplification and sequencing of 16S rDNA are dependent on the sequences already deposited in public databases [45]. At the end of successive sub culturing cycles, although with reduced richness and diversity, the consortia obtained are still highly complex, being composed of dominant as well as rare species. Despite the fact that rare microorganisms are important to ecosystem function, in our work we focused on the abundant taxa [46].

Taxonomic profile graphs at family level revealed the bacterial composition and dynamics of consortia across the six passages (Figs 2 and 3). Bacterial consortia tended to stabilize in terms of family composition after the fourth passage. A similar result was obtained by Yu et al. (2015) [27] in a lignin degrading microbial consortium enrichment study, where the consortium reached phylum-level stability after the third passage. In the work by Fang et al. (2018) [29], the microbial community of the ligninolytic consortia inoculated with samples of decaying tree trunks and soil close to the trunks reached stability in their genera composition after 6 days of cultivation. These data suggest that the taxonomic profile of consortia at different hierarchical levels may reach stability in terms of the composition at different times.

When observing the family composition of each consortium, it is noticeable that each consortium tends to acquire its own specific profile. The divergence in bacterial family composition among consortia indicates that the variables used during the enrichment process, such as temperature and substrate, changed the composition of initial bacteria in the consortia. Our results indicate that the type of lignin used as a carbon source in the enrichment experiment, Kraft lignin or lignin extracted by alkaline method, favored distinct groups of bacteria. At passage sixth, Kraft lignin favored families of the Proteobacteria phylum, while base-extracted lignin favored the Firmicutes phylum. Kraft lignin is a highly pure commercial lignin. As previously mentioned, during the manual process of lignin extraction by alkaline method, small amounts of carbohydrates may remain, and act as an alternative source, acting as a “start-up” carbon source for the degradation of polymers [47]. Certain groups of microorganisms require an easily metabolizable co-substrate for degradation of aromatic compounds, as in the case of lignin biodegradation [48, 49]. Alternative sources of carbon may be needed to promote Kraft lignin degradation due to its high molecular weight [50]. The literature points to some studies in which the genera *Lysinibacillus*, *Paenibacillus* and *Bacillus* from the phylum Firmicutes act on the degradation of Kraft lignin in the presence of 1% glucose and 0.5% peptone as an alternative source of carbon and nitrogen [50–52]. Therefore, the favoring of different families of Proteobacteria or Firmicutes according to the type of lignin used may be related to the need of the phylum Firmicutes for an alternative carbon source to start the process of lignin degradation. It is also possible that representatives of the Proteobacteria phylum are strong competitors favored in the presence of Kraft lignin, not allowing the colonization of the environment by species of the phylum Firmicutes. Also note that, although not predominant, bacteria from the Firmicutes phylum were present in the consortia fed with Kraft lignin, so it cannot be ruled out that the genera of the phylum Firmicutes present in our experiments can act on Kraft lignin in the absence of alternative carbon sources.

The phyla Bacteroidetes and Actinobacteria have also been identified in microbial consortia enriched to degrade lignin [10, 27, 30, 53]. However, only one representative of the phylum Actinobacteria was identified in the sixth passage of the consortia inoculated with Kraft lignin: Microbacteriaceae. The Microbacteriaceae family was also present in the enriched Lig Met consortium that used Kraft lignin as carbon source reported by Moraes et al. (2018) [10].

Some of the genera and families found in this work have previously been identified by their ligninolytic activity. In the enriched Lig Met consortium that used Kraft lignin isolated from black liquor obtained from delignification of sugarcane bagass, reported by Moraes et al. (2018) [10], it was possible to identify the classes Betaproteobacteria, Alphaproteobacteria, Gammaproteobacteria and members of the phyla Actinobacteria and Firmicutes. Similar to our results, Moraes et al. (2018) found that representatives of the phylum Proteobacteria were the most abundant in the consortium grown with Kraft lignin. Within the phylum Firmicutes that was also reported in this work, *Paenibacillus* from the phylum Firmicutes was one of the main genera identified. In our work, the genus *Paenibacillus* is one of the most abundant in the sixth passage of the enriched consortia that used lignin extracted by alkaline method as carbon source, but its relative abundance was less than 0.5% in the sixth passage of consortia enriched using Kraft lignin.

Another interesting fact is that the Flavobacteriaceae family of the order Flavobacteriales, Pseudomonadaceae of the order Pseudomonadales, Sphingobacteriaceae of the order Sphingobacteriales also have a great representation in the sixth passage of consortia inoculated with both soils. The Sphingobacteriales and Pseudomonadaceae were present in the sixth passage in three of four consortia inoculated with Miracle Growth (i.e., MG BE LIG 30°C, MG Kraft 30°C and 37°C (Fig 2A, 2C and 2D). In the consortia inoculated with backyard compost soil, the Pseudomonadaceae was present in two of four consortia (i.e., BY BE LIG 30°C and BY BE LIG 37°C (Fig 3A and 3B). The Flavobacteriaceae family was present in MG BE LIG 30°C, MG Kraft 30°C and BY Kraft 30°C (Figs 2A, 2C and 3C). These orders were also identified by Jiménez et al. (2016) [28] in the microbial consortium SG-M enriched to degrade maize straw that showed activity to degrade lignin.

Bacterial families of the classes Betaproteobacteria, Alphaproteobacteria, Gammaproteobacteria, Bacilli, Planococaceae were found in all consortia derived from MG and backyard compost soils, and these classes are frequently studied for presenting species capable of degrading lignin and its aromatic compounds [10]. Some genera of these classes have been reported as ligninolytic bacteria, namely *Ochrbactrum*, *Rhizobiales*, *Sphingobium*, *Lysinibacillus* and *Bacillus* [10, 54].

Analyzing the taxonomy data of the bacterial families of the microbial consortia MG BE lig 30°C and MG Kraft 30°C (Fig 2), the third passage proved to be different in terms of the composition of families from the rest of the passages. Note that in the third passage of the consortium MG BE lig 30°C, the families Pseudomonadaceae and Sphingomonadaceae of the phylum Protebacteria become dominant, while the families Bacillaceae and Planococcaceae decrease in terms of abundance (Fig 2A). The opposite occurs in the third passage of the consortium MG Kraft 30°C, where the families Bacillaceae and Planococcaceae become dominant while the families Pseudomonadaceae and Sphingomonadaceae decrease in terms of abundance (Fig 2C). It is also noticeable a change that occurs in the BE lig 37°C consortium, where the Bacillaceae family suddenly increases its relative abundance in the third pass (from 19% to 54%) and decreases the abundance in the fourth pass again to 21% (Fig 2B). The same occurred in relation to the taxonomy data of the bacterial families of the microbial consortia inoculated with backyard compost soil. In consortium BY BE lig 30°C, BY BE lig 37°C and BY Kraft 30°C in the third passage, the family Paenibacillaceae is high in abundance and then in the fourth passage it decreases significantly (Fig 3A, 3B and 3C). There are different hypothesis to explain this atypical data found in both experiments with MG and backyard compost soil: 1. this dynamic is due to the multiple interactions among the microbial community members including competition between species; 2. poor sample homogenization, which could lead to poor representation of the families present; 3. presence of spores of microorganisms that germinated during the enrichment. Some genera of the phylum Firmicutes have been studied extensively for their ability to form endospores [55–58]; and 4. presence of ciliated protozoa detected by sequencing the ITS region (S3 Table), which can feed on bacteria [59]. The activity of ciliates in microbial consortia can affect the dynamics of the bacterial community [10].

Venn diagrams (Fig 4) were useful to show information at the genus level, and provided insight about which genera were exclusive to certain conditions and which were the commonalities. Venn diagrams showed that the genus *Sphingobium* was present in three of the four consortia inoculated with MG soil and in the two of four consortia inoculated with backyard compost soil. This genus showed highest abundance in the enriched consortia that used Kraft lignin as carbon source (i.e., MG Kraft 30°C and BY Kraft 30°C). Previous works demonstrate that species of this genus present ligninolytic enzymes and pathways to catabolize the compounds generated in the degradation of lignin [60, 61]. In this study, this genus managed to persisted despite the difference in temperature and lignin variables, confirming what previous studies pointed out about its well-studied ligninolytic capacity.

The genus *Microvirga* represents 1% of the abundance of the MG-soil derived bacterial consortium in the sixth passage of that used base-extracted lignin and was grown at 37°C (i.e., MG BE lig 37°C) and this genus is also present in the sixth passage of the backyard compost soil derived consortia that used Kraft lignin and was grown at 30°C (i.e., Kraft Lig 30°C). A species in this genus isolated from nodules of the plant *Vicia alpestris*, *Microvirga ossetia*, had its genome completely sequenced and this allowed identification of a laccase [62]. This enzyme is part of the group of oxidoreductases, enzymes that are capable of degrading lignin [20, 21].

The *Aminobacter* genus of the Phyllobacteriaceae family is present in the microbial consortia MG Kraft 37°C and BY Kraft 37°C in the sixth passage (Figs 2, 3 and 4). Although no previous studies of enriched microbial consortia for lignin degradation have identified species of the genus *Aminobacter*, sequencing of the genome from a member of this genus indicates the presence of a ligninolytic enzyme, a laccase [63]. This representative of the genus in our experiments may have a role on the degradation of lignin, indicating the need for further characterization of the activities by bacteria of this genus.

For OTUs classified as Other or Unknown throughout this work, we were unable to assign the sequence to a taxon [45]. This result may indicate the lack of sequences in databases to compare the results obtained or it may indicate that for these OTUs the sequence presents errors and it was not possible to find homologous sequences in the databases. Nevertheless, OTUs classified in Other and Unknown should not be disregarded as, most likely, they represent groups not yet classified with ligninolytic activity.

The Venn diagram shows that the OTU at the genus level *Other 120* (family *Other 59*) of the order Burkholderiales appeared in all MG soil derived consortia having the greatest abundance in the consortium enriched to degrade Kraft lignin and grown at 37°C (Figs 2D and 4A). This genus (*Other* 120) appeared in all BY soil derived consortia as well, and had the highest abundance in the consortium BE Lig 37°C (Figs 2B and 4B). Despite the lack of taxonomic classification at the genus level, this OTU should not have its ligninolytic activity disregarded since species of this order have already been described in the literature acting on lignin degradation. As an example, the species *Burkholderia* sp. strain CCA53 of the order Burkholderiales was identified based on 16S rDNA gene sequencing and it could grow in culture medium with lignin monomers or alkali lignin as the only carbon source proved that this species expresses ligninolytic enzymes that allow lignin degradation [64]. This strain, in addition to degrading lignin, uses the released aromatic compounds as a carbon source.

For the genera Other 86 present in the MG BE Lig 30°C and BY BE Lig 37°C consortia (Fig 4), we were unable to assign the taxonomic classification at any hierarchical level (Figs 2 and 3). It can be inferred for this genus that the type of lignin used in the enrichment process was an important variable since this genus remains abundant only in consortia enriched with lignin extracted by alkaline method. On the other hand, this genus seems to support temperature variation. Despite appearing with relative abundance higher than 1% in the sixth passage of the referred consortia, its identity remains unknown.

### 4.3. Influence of temperature and type of lignin in the selection of bacteria

When compared to fungi, bacterial ligninolytic enzymes have a lower redox potential, so structural differences in lignin would lead to the need for different enzymes to degrade different parts of lignin [65]. It is believed that for the microbial consortia analyzed in this work, the bacteria would be acting in cooperation to degrade different fractions of lignin. It is known that different types of pre-treatment can give rise to different three-dimensional structures of lignin and it is known that the process of biodegradation of lignin by microorganisms depends on the type of lignin structure [19, 65–68]. Therefore, the structural differences between Kraft lignin and lignin extracted by alkaline method may be sufficient to select different groups of microorganisms capable of degrading them.

In this work, Bray-Curtis dissimilarities were visualized with NMDS. Fig 5 showed grouping according to the type of carbon source, as well as grouping of MG- and backyard (BY) compost-derived consortia using Kraft lignin as carbon source. To test the significance of these results, PERMANOVA testing was performed and the amount of variance correlated with each variable studied (type of soil, passage, type of lignin and temperature) was obtained. PERMANOVA is a method that uses permutation for non‐parametric multivariate analysis of variance. The type of lignin, base-extracted or Kraft lignin, was able to best explain variation and corresponded to approximately 32% of the variation observed. Therefore, despite the temperature being one of the important physical factors that affects microbial growth, the variation of 7°C between the cultivation temperatures was not as important as the type of carbon source to differentiate the consortia. Although microorganisms have an optimal growth temperature, but they can also grow at a range of temperatures [69]. As mentioned previously, base-extracted lignin may have been less pure, and therefore may be metabolized by bacteria that are able to use lignin as well as other alternative contaminating carbon source. On the other hand, Kraft lignin would select for groups of bacteria that can specifically metabolize this type of lignin. Therefore, it would be expected that the taxa driving the Kraft lignin cluster in Fig 5 would be more stringent in their ability to use lignin as a carbon source.

## 5. Conclusions

Most works about degradation of lignin focus on fungi. To increase our knowledge on ligninolytic bacteria, we used the powerful method of culture enrichment to select from highly diverse soil microorganisms the ones most adapted to using lignin as a carbon source. To the best of our knowledge our work has used the most variables to obtain the consortia (two sources of inoculum, two cultivation temperatures and two types of lignin). High-throughput 16S rRNA gene sequencing was used to assess the bacterial community structure during the enrichment process. Consistent with a selection process, diversity indexes showed a decrease in bacterial richness as well as diversity in consortia at the end of six enrichment cycles. Analysis of bacterial composition over 2-week enrichment cycles indicated that stability was achieved beyond the fourth cycle. Although the focus of the present work was bacteria, the presence of ciliates in the consortia may have influenced the dynamics of the bacterial community in the microbial consortia. The main known bacterial genera selected to use lignin as a carbon source were *Altererythrobacter*, *Aminobacter*, *Bacillus*, *Burkholderia*, *Lysinibacillus*, *Microvirga*, *Mycobacterium*, *Ochrobactrum*, *Paenibacillus*, *Pseudomonas*, *Pseudoxanthomonas*, *Rhizobiales* and. *Sphingobium*. There were also OTUs classified as Other and Unknown indicating the presence of bacteria not yet resolved taxonomically that may play a direct or indirect role in lignin degradation. These bacterial genera can be of particular interest for studying lignin degradation and utilization as well as for the development of lignin-related biotechnology applications.

## Supporting information

S1 Table Diversity indexes for the consortia in the original MG compost soil (0P) and six enrichment cycles or passages (1P, 2P, 3P, 4P, 5P and 6P) using either base-extracted of Kraft lignin as carbon source and cultivated either at 30°C or 37°C. (DOCX)

S2 Table Diversity indexes for the consortia in the original backyard (BY) compost soil (0P) and six enrichment cycles or passages (1P, 2P, 3P, 4P, 5P and 6P) using either base-extracted of Kraft lignin as carbon source and cultivated either at 30°C or 37°C. (DOCX)

S3 Table Relative abundance (%) of phylum ciliophora for the consortia in the original backyard (BY) compost soil (0P) and six enrichment cycles or passages (1P, 2P, 3P, 4P, 5P and 6P) using either base-extracted of Kraft lignin as carbon source and cultivated either at 30°C or 37°C. Data based on sequencing of the ITS region*. *Primers used for amplification were FW (ITS9): 5’ GAA CGC AGC RAA IIG YGA 3’ and RV (ITS4): 5’ TCC TCC GCT TAT TGA TAT GC 3’ [34].
(DOCX)

S4 Table Percentage of each bacterial group at family level depicted in Fig 2 present in consortia obtained from MG soil (MG) over successive passages (0, 1, 2, 3, 4, 5, 6) in enrichment experiment using M9 medium containing either base-extracted lignin, BE, or Kraft lignin, at two temperatures. 30°C and 37°C. For the families classified as Other and Unknown, we were unable to obtain the taxonomic affiliation at the family level, so the closest previous hierarchical level is provided.
(DOCX)

S5 Table Percentage of each bacterial group at family level depicted in Fig 2 present in consortia obtained from backyard soil (BY) over successive passages (0, 1, 2, 3, 4, 5, 6) in enrichment experiment using M9 medium containing either base-extracted lignin, BE, or Kraft lignin, at two temperatures. 30°C and 37°C. For the families classified as Other and Unknown, we were unable to obtain the taxonomic affiliation at the family level, so the closest previous hierarchical level is provided.
(DOCX)

S1 File Variance of the Bray Curtis dissimilarities matrix associated with substrate (base extracted and Kraft lignin), temperature (30°C and 37°C), Passage (0 to 6) and soil (MG and BY). Bray Curtis matrix PERMANOVA analysis was performed using the adonis function of vegan package in R.
(DOCX)

S2 File Taxa driving clusters (p-value < = 0.0001) based on substrates (lignin type: base extracted and Kraft lignin) observed in NMDS plot (Fig 5). Vectors were found using the envfit function of vegan package in R performing 10,000 permutations.
(XLSX)

S1 Data Supplementary data for Fig 4. (DOCX)
