# Supplementary material for: Bacterial diversity dynamics in microbial consortia selected for lignin utilization
Source: PLoS One. 2021 Sep 13;16(9):e0255083. doi: 10.1371/journal.pone.0255083 (PMC8437272; doi:10.1371/journal.pone.0255083)
Supplement: S1 Table — (DOCX) [file pone.0255083.s001.docx]

**S1 Table.** Diversity indexes for the consortia in the original MG compost soil (0P) and six enrichment cycles or passages (1P, 2P, 3P, 4P, 5P and 6P) using either base-extracted of Kraft lignin as carbon source and cultivated either at 30 ºC or 37 ºC.

| **Passage** | **Substrate** | **Temperature** | **Observed OTUs Ave.** | **Good's Coverage** | **Chao 1** | **PD_whole_tree** | **Shannon** | **1-Simpson** |
| --- | --- | --- | --- | --- | --- | --- | --- | --- |
| **0P** | - | - | 307 | 100% | 346.723 | 32.327 | 4.091 | 0.861 |
| **1P** | BE Lig | 30° C | 348 | 100% | 389.427 | 30.405 | 4.936 | 0.905 |
| **2P** |  |  | 217 | 100% | 249.218 | 19.690 | 2.883 | 0.757 |
| **3P** |  |  | 225 | 100% | 246.670 | 19.917 | 3.866 | 0.811 |
| **4P** |  |  | 214 | 100% | 235.051 | 19.773 | 2.785 | 0.675 |
| **5P** |  |  | 235 | 100% | 267.531 | 21.678 | 3.559 | 0.810 |
| **6P** |  |  | 209 | 100% | 227.927 | 22.425 | 3.467 | 0.803 |
| **1P** |  | 37° C | 283 | 100% | 308.973 | 20.875 | 4.680 | 0.904 |
| **2P** |  |  | 199 | 100% | 212.241 | 20.494 | 3.882 | 0.850 |
| **3P** |  |  | 178 | 100% | 202.986 | 17.178 | 3.268 | 0.695 |
| **4P** |  |  | 178 | 100% | 195.789 | 19.635 | 2.770 | 0.667 |
| **5P** |  |  | 179 | 100% | 203.204 | 19.369 | 3.031 | 0.725 |
| **6P** |  |  | 171 | 100% | 184.670 | 18.653 | 2.662 | 0.682 |
| **1P** | Kraft | 30° C | 286 | 100% | 320.768 | 27.450 | 4.476 | 0.908 |
| **2P** |  |  | 164 | 100% | 175.741 | 15.826 | 3.129 | 0.754 |
| **3P** |  |  | 223 | 100% | 244.119 | 22.928 | 4.182 | 0.885 |
| **4P** |  |  | 139 | 100% | 153.953 | 12.918 | 3.011 | 0.676 |
| **5P** |  |  | 153 | 100% | 164.780 | 13.208 | 3.327 | 0.739 |
| **6P** |  |  | 143 | 100% | 158.112 | 12.750 | 3.428 | 0.766 |
| **1P** |  | 37° C | 223 | 100% | 278.724 | 23.329 | 3.977 | 0.885 |
| **2P** |  |  | 134 | 100% | 156.036 | 13.494 | 3.660 | 0.836 |
| **3P** |  |  | 124 | 100% | 138.712 | 13.702 | 3.669 | 0.856 |
| **4P** |  |  | 117 | 100% | 133.507 | 9.890 | 4.081 | 0.905 |
| **5P** |  |  | 108 | 100% | 125.017 | 10.934 | 3.975 | 0.897 |
| **6P** |  |  | 107 | 100% | 114.618 | 10.081 | 4.104 | 0.904 |
