# Supplementary material for: Bacterial diversity dynamics in microbial consortia selected for lignin utilization
Source: PLoS One. 2021 Sep 13;16(9):e0255083. doi: 10.1371/journal.pone.0255083 (PMC8437272; doi:10.1371/journal.pone.0255083)
Supplement: S2 Table — (DOCX) [file pone.0255083.s002.docx]

**S2 Table.** Diversity indexes for the consortia in the original backyard (BY) compost soil (0P) and six enrichment cycles or passages (1P, 2P, 3P, 4P, 5P and 6P) using either base-extracted of Kraft lignin as carbon source and cultivated either at 30 ºC or 37 ºC.

| **Passage** | **Substrate** | **Temperature** | **Observed_OTUs Ave.** | **Good's Coverage** | **Chao 1** | **PD_whole_tree** | **Shannon** | **1-Simpson** |
| --- | --- | --- | --- | --- | --- | --- | --- | --- |
| **0P** | - | - | 1,061 | 99% | 1,175.026 | 72.810 | 7.991 | 0.990 |
| **1P** | BE Lig | 30° C | 504 | 99% | 655.808 | 38.405 | 4.500 | 0.875 |
| **2P** |  |  | 323 | 100% | 356.523 | 26.957 | 4.612 | 0.875 |
| **3P** |  |  | 276 | 100% | 294.483 | 26.277 | 4.477 | 0.873 |
| **4P** |  |  | 238 | 100% | 266.543 | 23.038 | 3.790 | 0.859 |
| **5P** |  |  | 219 | 100% | 236.585 | 22.760 | 3.677 | 0.832 |
| **6P** |  |  | 230 | 100% | 248.303 | 22.722 | 4.075 | 0.885 |
| **1P** |  | 37° C | 373 | 99% | 496.191 | 29.310 | 3.910 | 0.821 |
| **2P** |  |  | 191 | 100% | 228.976 | 18.688 | 3.006 | 0.750 |
| **3P** |  |  | 192 | 100% | 215.955 | 20.079 | 2.906 | 0.699 |
| **4P** |  |  | 151 | 100% | 167.743 | 17.070 | 3.027 | 0.665 |
| **5P** |  |  | 171 | 100% | 178.593 | 15.579 | 2.993 | 0.662 |
| **6P** |  |  | 154 | 100% | 173.545 | 18.517 | 3.770 | 0.839 |
| **1P** | Kraft | 30° C | 564 | 99% | 661.095 | 41.242 | 5.486 | 0.938 |
| **2P** |  |  | 255 | 100% | 332.862 | 23.870 | 5.390 | 0.958 |
| **3P** |  |  | 261 | 100% | 279.804 | 25.870 | 3.769 | 0.793 |
| **4P** |  |  | 182 | 100% | 200.825 | 18.748 | 4.737 | 0.910 |
| **5P** |  |  | 165 | 100% | 172.955 | 18.240 | 4.579 | 0.905 |
| **6P** |  |  | 171 | 100% | 185.470 | 15.421 | 4.930 | 0.934 |
| **1P** |  | 37° C | 404 | 100% | 483.033 | 34.034 | 4.929 | 0.941 |
| **2P** |  |  | 148 | 100% | 186.544 | 12.859 | 4.537 | 0.936 |
| **3P** |  |  | 112 | 100% | 120.386 | 13.932 | 4.402 | 0.930 |
| **4P** |  |  | 106 | 100% | 123.598 | 12.919 | 4.042 | 0.897 |
| **5P** |  |  | 105 | 100% | 115.854 | 13.218 | 3.535 | 0.802 |
| **6P** |  |  | 101 | 100% | 109.451 | 13.352 | 3.500 | 0.806 |
