# Supplementary material for: Bacterial diversity dynamics in microbial consortia selected for lignin utilization
Source: PLoS One. 2021 Sep 13;16(9):e0255083. doi: 10.1371/journal.pone.0255083 (PMC8437272; doi:10.1371/journal.pone.0255083)
Supplement: S3 Table — Data based on sequencing of the ITS region*. *Primers used for amplification were FW (ITS9): 5’ GAA CGC AGC RAA IIG YGA 3’ and RV (ITS4): 5’ TCC TCC GCT TAT TGA TAT GC 3’ [34]. (DOCX) [file pone.0255083.s003.docx]

**S3 Table.** Relative abundance (%) of phylum Ciliophora for the consortia in the original backyard (BY) compost soil (0P) and six enrichment cycles or passages (1P, 2P, 3P, 4P, 5P and 6P) using either base-extracted of Kraft lignin as carbon source and cultivated either at 30 ºC or 37 ºC. Data based on sequencing of the ITS region*.
