# Supplementary material for: Bacterial diversity dynamics in microbial consortia selected for lignin utilization
Source: PLoS One. 2021 Sep 13;16(9):e0255083. doi: 10.1371/journal.pone.0255083 (PMC8437272; doi:10.1371/journal.pone.0255083)
Supplement: S4 Table — 30°C and 37°C. For the families classified as Other and Unknown, we were unable to obtain the taxonomic affiliation at the family level, so the closest previous hierarchical level is provided. (DOCX) [file pone.0255083.s004.docx]

**S4 Table.** Percentage of each bacterial group at family level depicted in figure 2 present in consortia obtained from MG soil (MG) over successive passages (0, 1, 2, 3, 4, 5, 6) in enrichment experiment using M9 medium containing either base-extracted lignin, BE, or Kraft lignin, at two temperatures. 30 ºC and 37 ºC. For the families classified as Other and Unknown, we were unable to obtain the taxonomic affiliation at the family level, so the closest previous hierarchical level is provided.

| **MG BE 30°C** | | | | | | | |
| --- | --- | --- | --- | --- | --- | --- | --- |
| **Passage** | | | | | | | |
| **Family** | **0** | **1** | **2** | **3** | **4** | **5** | **6** |
| Acetobacteraceae | 0.00 | 0.00 | 0.00 | 0.00 | 0.00 | 0.00 | 0.00 |
| Alcanivoracaceae | 4.61 | 0.050 | 0.06 | 0.00 | 0.02 | 0.02 | 0.02 |
| Bacillaceae | 0.10 | 22.12 | 13.80 | 4.19 | 3.70 | 5.34 | 8.61 |
| Beijerinckiaceae | 0.00 | 0.360 | 0.050 | 1.05 | 0.21 | 0.82 | 0.41 |
| Brucellaceae | 0.02 | 1.07 | 0.18 | 3.41 | 0.15 | 0.45 | 0.54 |
| Burkholderiaceae | 0.00 | 0.00 | 0.00 | 0.00 | 0.00 | 0.00 | 0.00 |
| Chitinophagaceae | 21.30 | 2.37 | 0.80 | 2.26 | 0.86 | 0.51 | 0.91 |
| Cryomorphaceae | 4.61 | 0.52 | 0.28 | 0.02 | 0.10 | 0.13 | 0.25 |
| Erythrobacteraceae | 0.01 | 2.17 | 0.23 | 1.94 | 0.36 | 0.31 | 0.61 |
| Flavobacteriaceae | 0.00 | 10.46 | 34.37 | 3.95 | 17.75 | 11.34 | 13.40 |
| Hyphomicrobiaceae | 0.29 | 1.87 | 0.10 | 1.16 | 0.12 | 0.35 | 0.45 |
| Microbacteriaceae | 2.16 | 2.27 | 0.22 | 1.51 | 0.16 | 0.21 | 0.40 |
| Nocardioidaceae | 2.81 | 0.75 | 0.03 | 0.07 | 0.01 | 0.01 | 0.02 |
| Other 9 - Order: Sphingobacteriales | 0.36 | 0.45 | 6.82 | 0.75 | 6.40 | 1.86 | 3.47 |
| Other 18 - Phyum: Other | 0.42 | 0.62 | 0.28 | 0.22 | 0.56 | 0.97 | 1.62 |
| Other 20 - Order: Planctomycetales | 0.06 | 1.98 | 0.61 | 0.18 | 1.29 | 3.52 | 2.86 |
| Other 23 - Order: Rhodospirillales | 0.03 | 0.06 | 0.00 | 1.57 | 0.00 | 0.00 | 0.00 |
| Other 24 - Order: Sphingomonadales | 0.00 | 0.00 | 0.00 | 0.00 | 0.00 | 0.00 | 0.00 |
| Other 25 - Order: Burkholderiales | 14.54 | 3.87 | 1.63 | 5.92 | 1.36 | 2.27 | 2.38 |
| Other 26 - Class: Betaproteobacteria | 0.27 | 0.60 | 0.43 | 4.01 | 0.47 | 0.56 | 0.64 |
| Paenibacillaceae | 0.29 | 1.15 | 0.94 | 0.67 | 5.51 | 27.51 | 17.57 |
| Phyllobacteriaceae | 0.00 | 0.00 | 0.00 | 0.00 | 0.00 | 0.000 | 0.000 |
| Planctomycetaceae | 0.00 | 0.00 | 0.00 | 0.00 | 0.00 | 0.000 | 0.000 |
| Planococcaceae | 0.03 | 18.40 | 31.95 | 6.52 | 53.50 | 34.89 | 37.81 |
| Promicromonosporaceae | 0.00 | 0.00 | 0.00 | 0.00 | 0.00 | 0.00 | 0.00 |
| Pseudomonadaceae | 0.01 | 2.57 | 0.74 | 41.27 | 0.40 | 1.57 | 0.52 |
| Rhodobacteraceae | 0.00 | 0.00 | 0.12 | 0.32 | 2.21 | 1.50 | 1.12 |
| Sphingobacteriaceae | 0.52 | 1.17 | 0.68 | 28.30 | 0.51 | 0.41 | 0.64 |
| Sphingomonadaceae | 0.73 | 3.38 | 0.27 | 10.42 | 0.54 | 0.27 | 0.68 |
| Streptomycetaceae | 2.73 | 0.16 | 0.00 | 0.00 | 0.00 | 0.00 | 0.00 |
| Unknown_Family_1002292 Phylum: Chloroflexi | 0.00 | 0.00 | 0.00 | 0.00 | 0.00 | 0.00 | 0.00 |
| Vulgatibacteraceae | 0.00 | 0.00 | 0.00 | 0.00 | 0.00 | 0.00 | 0.00 |
| Xanthomonadaceae | 30.57 | 4.98 | 0.57 | 0.43 | 0.13 | 0.25 | 0.29 |
| **MG BE 37°C** | | | | | | | |
| **Passage** | | | | | | | |
| **Family** | **0** | **1** | **2** | **3** | **4** | **5** | **6** |
| Acetobacteraceae | 0.00 | 0.00 | 0.00 | 0.00 | 0.00 | 0.00 | 0.00 |
| Alcanivoracaceae | 4.60 | 0.00 | 0.00 | 0.00 | 0.00 | 0.00 | 0.00 |
| Bacillaceae | 0.10 | 2.87 | 19.88 | 54.41 | 21.71 | 28.99 | 32.65 |
| Beijerinckiaceae | 0.00 | 1.25 | 1.28 | 0.95 | 0.19 | 0.20 | 0.17 |
| Brucellaceae | 0.01 | 0.12 | 0.49 | 1.26 | 0.19 | 0.09 | 0.08 |
| Burkholderiaceae | 0.00 | 0.00 | 0.00 | 0.00 | 0.00 | 0.00 | 0.00 |
| Chitinophagaceae | 21.30 | 0.01 | 0.13 | 0.01 | 0.03 | 0.03 | 0.07 |
| Cryomorphaceae | 4.60 | 0.00 | 0.00 | 0.00 | 0.00 | 0.00 | 0.00 |
| Erythrobacteraceae | 0.00 | 0.66 | 0.69 | 0.44 | 1.37 | 0.95 | 0.93 |
| Flavobacteriaceae | 0.00 | 0.00 | 0.00 | 0.00 | 0.00 | 0.00 | 0.00 |
| Hyphomicrobiaceae | 0.29 | 1.50 | 0.76 | 0.99 | 0.39 | 0.37 | 0.30 |
| Microbacteriaceae | 2.16 | 2.02 | 1.13 | 0.37 | 0.18 | 0.31 | 0.23 |
| Nocardioidaceae | 2.81 | 0.42 | 0.01 | 0.08 | 0.01 | 0.01 | 0.02 |
| Other 9 - Ordem: Sphingobacteriales | 0.00 | 0.00 | 0.00 | 0.00 | 0.00 | 0.00 | 0.00 |
| Other 18 - Filo: Other | 0.42 | 1.26 | 4.46 | 5.05 | 1.04 | 2.07 | 0.97 |
| Other 20 - Ordem: Planctomycetales | 0.06 | 1.23 | 0.18 | 0.24 | 0.23 | 0.33 | 0.28 |
| Other 23 - Ordem: Rhodospirillales | 0.00 | 0.00 | 0.00 | 0.00 | 0.00 | 0.00 | 0.00 |
| Other 24 - Ordem: Sphingomonadales | 0.00 | 0.00 | 0.00 | 0.00 | 0.00 | 0.00 | 0.00 |
| Other 25 - Ordem: Burkholderiales | 14.54 | 3.72 | 9.21 | 9.55 | 1.11 | 1.28 | 3262 |
| Other 26 - Classe Betaproteobacteria | 0.00 | 0.00 | 0.00 | 0.00 | 0.00 | 0.00 | 0.00 |
| Paenibacillaceae | 0.29 | 2.24 | 6.43 | 11.22 | 8.27 | 7.30 | 3.89 |
| Phyllobacteriaceae | 0.36 | 0.91 | 0.96 | 0.73 | 1.27 | 0.40 | 0.65 |
| Planctomycetaceae | 0.31 | 13.98 | 3.74 | 1.70 | 1.05 | 0.84 | 0.78 |
| Planococcaceae | 0.03 | 1.48 | 32.64 | 1.19 | 53.43 | 44.26 | 46.59 |
| Promicromonosporaceae | 0.11 | 1.06 | 0.23 | 0.00 | 0.00 | 0.00 | 0.00 |
| Pseudomonadaceae | 0.00 | 0.00 | 0.00 | 0.00 | 0.00 | 0.00 | 0.00 |
| Rhodobacteraceae | 0.00 | 0.00 | 0.00 | 0.00 | 0.00 | 0.00 | 0.00 |
| Sphingobacteriaceae | 0.00 | 0.00 | 0.00 | 0.00 | 0.00 | 0.00 | 0.00 |
| Sphingomonadaceae | 0.72 | 7.43 | 2.73 | 1.79 | 0.80 | 1.91 | 1.51 |
| Streptomycetaceae | 2.73 | 0.24 | 0.00 | 0.00 | 0.00 | 0.00 | 0.00 |
| Unknown_Family_1002292 | 0.00 | 2.59 | 0.33 | 0.63 | 0.21 | 0.27 | 0.13 |
| Vulgatibacteraceae | 0.00 | 6.89 | 1.84 | 0.29 | 0.86 | 1.21 | 0.98 |
| Xanthomonadaceae | 30.57 | 7.81 | 5.76 | 0.17 | 0.00 | 0.00 | 0.00 |
| **MG Kraft 30°C** | | | | | | | |
| **Passage** | | | | | | | |
| **Family** | **0** | **1** | **2** | **3** | **4** | **5** | **6** |
| Acetobacteraceae | 0.00 | 0.00 | 0.00 | 0.00 | 0.00 | 0.00 | 0.00 |
| Alcanivoracaceae | 4.60 | 0.02 | 0.00 | 0.00 | 0.00 | 0.00 | 0.00 |
| Bacillaceae | 0.10 | 0.05 | 0.00 | 13.83 | 0.00 | 0.00 | 0.00 |
| Beijerinckiaceae | 0.00 | 0.34 | 0.77 | 1.10 | 1.11 | 1.03 | 1.04 |
| Brucellaceae | 0.01 | 1.95 | 4.02 | 3.34 | 4.06 | 3.92 | 3.93 |
| Burkholderiaceae | 0.02 | 4.48 | 0.51 | 0.52 | 0.63 | 2.64 | 3.59 |
| Chitinophagaceae | 21.30 | 27.40 | 20.48 | 1.60 | 3.05 | 1.88 | 2.34 |
| Cryomorphaceae | 4.61 | 6.41 | 0.00 | 0.02 | 0.00 | 0.00 | 0.00 |
| Erythrobacteraceae | 0.00 | 0.86 | 1.27 | 2.18 | 2.25 | 2.75 | 2.87 |
| Flavobacteriaceae | 0.00 | 3.60 | 2.36 | 5.83 | 0.90 | 0.66 | 0.84 |
| Hyphomicrobiaceae | 0.29 | 1.12 | 0.74 | 1.39 | 1.65 | 2.33 | 1.86 |
| Microbacteriaceae | 2.16 | 2.45 | 0.81 | 1.60 | 1.54 | 1.07 | 1.01 |
| Nocardioidaceae | 2.81 | 0.36 | 0.08 | 0.11 | 0.05 | 0.11 | 0.11 |
| Other 9 - Ordem: Sphingobacteriales | 0.36 | 0.46 | 0.03 | 3.14 | 0.00 | 0.00 | 0.00 |
| Other 18 - Filo: Other | 0.00 | 0.00 | 0.00 | 0.00 | 0.00 | 0.00 | 0.00 |
| Other 20 - Ordem: Planctomycetales | 0.00 | 0.00 | 0.00 | 0.00 | 0.00 | 0.00 | 0.00 |
| Other 23 - Ordem: Rhodospirillales | 0.02 | 0.06 | 0.28 | 0.65 | 2.30 | 3.10 | 6.22 |
| Other 24 - Ordem: Sphingomonadales | 0.00 | 0.00 | 0.00 | 0.00 | 0.00 | 0.00 | 0.00 |
| Other 25 - Ordem: Burkholderiales | 14.54 | 26.06 | 6.19 | 3.81 | 7.74 | 10.00 | 8.84 |
| Other 26 - Classe Betaproteobacteria | 0.27 | 0.90 | 2.07 | 2.06 | 2.31 | 2.33 | 1.96 |
| Paenibacillaceae | 0.29 | 0.19 | 0.04 | 1.77 | 0.02 | 0.00 | 0.00 |
| Phyllobacteriaceae | 0.00 | 0.00 | 0.00 | 0.00 | 0.00 | 0.00 | 0.00 |
| Planctomycetaceae | 0.00 | 0.00 | 0.00 | 0.00 | 0.00 | 0.00 | 0.00 |
| Planococcaceae | 0.03 | 0.04 | 0.00 | 21.69 | 0.00 | 0.00 | 0.00 |
| Promicromonosporaceae | 0.00 | 0.00 | 0.00 | 0.00 | 0.00 | 0.00 | 0.00 |
| Pseudomonadaceae | 0.00 | 7.66 | 44.22 | 20.22 | 55.81 | 49.48 | 46.35 |
| Rhodobacteraceae | 0.00 | 0.00 | 0.00 | 0.00 | 0.00 | 0.00 | 0.00 |
| Sphingobacteriaceae | 0.52 | 1.81 | 2.86 | 1.68 | 2.64 | 2.63 | 3.28 |
| Sphingomonadaceae | 0.72 | 4.26 | 8.03 | 6.54 | 8.84 | 9.81 | 10.60 |
| Streptomycetaceae | 2.73 | 0.06 | 0.00 | 0.00 | 0.00 | 0.00 | 0.00 |
| Unknown_Family_1002292 | 0.00 | 2.59 | 0.33 | 0.63 | 0.21 | 0.27 | 0.13 |
| Vulgatibacteraceae | 0.00 | 6.89 | 1.84 | 0.29 | 0.86 | 1.21 | 0.98 |
| Xanthomonadaceae | 30.57 | 0.62 | 0.10 | 0.49 | 0.56 | 0.27 | 0.31 |
| **MG Kraft 37°C** | | | | | | | |
| **Passage** | | | | | | | |
| **Family** | **0** | **1** | **2** | **3** | **4** | **5** | **6** |
| Acetobacteraceae | 0.14 | 0.19 | 1.02 | 1.71 | 1.87 | 2.42 | 1.54 |
| Alcanivoracaceae | 4.60 | 0.00 | 0.00 | 0.00 | 0.00 | 0.00 | 0.00 |
| Bacillaceae | 0.000 | 0.00 | 0.00 | 0.00 | 0.00 | 0.00 | 0.00 |
| Beijerinckiaceae | 0.00 | 0.56 | 1.89 | 1.21 | 1.74 | 1.06 | 1.33 |
| Brucellaceae | 0.01 | 10.51 | 5.05 | 14.27 | 12.94 | 19.31 | 17.30 |
| Burkholderiaceae | 0.02 | 5.89 | 0.04 | 0.00 | 0.00 | 0.00 | 0.00 |
| Chitinophagaceae | 21.30 | 21.24 | 0.03 | 0.00 | 0.00 | 0.00 | 0.00 |
| Cryomorphaceae | 4.60 | 0.00 | 0.00 | 0.00 | 0.00 | 0.00 | 0.00 |
| Erythrobacteraceae | 0.00 | 2.58 | 8.45 | 5.74 | 6.02 | 3.97 | 4.93 |
| Flavobacteriaceae | 0.00 | 0.00 | 0.00 | 0.00 | 0.00 | 0.00 | 0.00 |
| Hyphomicrobiaceae | 0.29 | 2.10 | 1.86 | 2.02 | 3.43 | 3.89 | 3.82 |
| Microbacteriaceae | 2.16 | 1.75 | 4.12 | 5.16 | 7.71 | 6.70 | 6.30 |
| Nocardioidaceae | 2.81 | 0.33 | 0.16 | 0.12 | 0.11 | 0.08 | 0.11 |
| Other 9 - Ordem: Sphingobacteriales | 0.00 | 0.00 | 0.00 | 0.00 | 0.00 | 0.00 | 0.00 |
| Other 18 - Filo: Other | 0.42 | 10.58 | 0.01 | 0.01 | 0.06 | 0.32 | 0.14 |
| Other 20 - Ordem: Planctomycetales | 0.00 | 0.00 | 0.00 | 0.00 | 0.00 | 0.00 | 0.00 |
| Other 23 - Ordem: Rhodospirillales | 0.00 | 0.00 | 0.00 | 0.00 | 0.00 | 0.00 | 0.00 |
| Other 24 - Ordem: Sphingomonadales | 0.00 | 0.96 | 6.02 | 4.51 | 6.55 | 4.73 | 5.38 |
| Other 25 - Ordem: Burkholderiales | 14.54 | 12.79 | 15.70 | 12.23 | 14.01 | 13.38 | 18.02 |
| Other 26 - Classe Betaproteobacteria | 0.27 | 1.97 | 5.41 | 0.45 | 0.36 | 0.25 | 0.21 |
| Paenibacillaceae | 0.29 | 1.20 | 0.96 | 0.99 | 0.81 | 0.83 | 0.64 |
| Phyllobacteriaceae | 0.36 | 0.19 | 2.58 | 9.39 | 9.87 | 14.29 | 10.33 |
| Planctomycetaceae | 0.00 | 0.00 | 0.00 | 0.00 | 0.00 | 0.00 | 0.00 |
| Planococcaceae | 0.00 | 0.00 | 0.00 | 0.00 | 0.00 | 0.00 | 0.00 |
| Promicromonosporaceae | 0.11 | 0.18 | 1.10 | 0.87 | 1.48 | 2.43 | 50.4 |
| Pseudomonadaceae | 0.06 | 18.83 | 34.90 | 30.42 | 18.72 | 13.85 | 11.06 |
| Rhodobacteraceae | 0.00 | 0.00 | 0.00 | 0.00 | 0.00 | 0.00 | 0.00 |
| Sphingobacteriaceae | 0.52 | 0.64 | 1.05 | 0.27 | 0.14 | 0.00 | 0.24 |
| Sphingomonadaceae | 0.72 | 1.59 | 4.37 | 5.57 | 7.40 | 5.55 | 5.18 |
| Streptomycetaceae | 2.73 | 0.07 | 0.00 | 0.00 | 0.00 | 0.00 | 0.00 |
| Unknown_Family_1002292 | 0.00 | 0.00 | 0.00 | 0.00 | 0.00 | 0.00 | 0.00 |
| Vulgatibacteraceae | 0.00 | 0.00 | 0.00 | 0.00 | 0.00 | 0.00 | 0.00 |
| Xanthomonadaceae | 30.57 | 0.40 | 0.95 | 0.77 | 0.84 | 0.61 | 0.73 |
