# Supplementary material for: Bacterial diversity dynamics in microbial consortia selected for lignin utilization
Source: PLoS One. 2021 Sep 13;16(9):e0255083. doi: 10.1371/journal.pone.0255083 (PMC8437272; doi:10.1371/journal.pone.0255083)
Supplement: S5 Table — 30°C and 37°C. For the families classified as Other and Unknown, we were unable to obtain the taxonomic affiliation at the family level, so the closest previous hierarchical level is provided. (DOCX) [file pone.0255083.s005.docx]

**S5 Table.** Percentage of each bacterial group at family level depicted in figure 2 present in consortia obtained from backyard soil (BY) over successive passages (0, 1, 2, 3, 4, 5, 6) in enrichment experiment using M9 medium containing either base-extracted lignin, BE, or Kraft lignin, at two temperatures. 30 ºC and 37 ºC. For the families classified as Other and Unknown, we were unable to obtain the taxonomic affiliation at the family level, so the closest previous hierarchical level is provided.

| **BE 30°C** | | | | | | | | |
| --- | --- | --- | --- | --- | --- | --- | --- | --- |
| **Passage** | | | | | | | | |
| **Family** | **0** | **1** | **2** | **3** | **4** | **5** | **6** |  |
| Bacillaceae | 6.15 | 33.98 | 38.17 | 27.88 | 56.78 | 56.80 | 28.63 |  |
| Beijerinckiaceae | 0.00 | 0.31 | 0.10 | 0.08 | 3.58 | 0.50 | 1.36 |  |
| BIrii41 | 3.24 | 0.00 | 0.00 | 0.00 | 0.00 | 0.00 | 0.00 |  |
| Bradyrhizobiaceae | 0.33 | 1.30 | 0.22 | 0.14 | 0.07 | 0.09 | 0.15 |  |
| Brucellaceae | 0.04 | 0.53 | 0.11 | 0.14 | 0.89 | 1.95 | 2.84 |  |
| Caulobacteraceae | 1.24 | 0.62 | 0.03 | 0.01 | 0.04 | 0.11 | 0.29 |  |
| Cellvibrionaceae | 2.28 | 0.00 | 0.00 | 0.00 | 0.00 | 0.00 | 0.00 |  |
| Chitinophagaceae | 3.81 | 0.10 | 0.03 | 0.03 | 0.00 | 0.00 | 0.01 |  |
| Comamonadaceae | 2.50 | 0.19 | 0.02 | 0.02 | 0.14 | 0.05 | 0.05 |  |
| Cytophagaceae | 6.71 | 0.02 | 0.00 | 0.00 | 0.00 | 0.00 | 0.00 |  |
| Fibrobacteraceae | 2.68 | 0.00 | 0.00 | 0.00 | 0.00 | 0.00 | 0.00 |  |
| Flavobacteriaceae | 1.32 | 0.00 | 0.00 | 0.00 | 0.00 | 0.00 | 0.00 |  |
| Hyphomicrobiaceae | 1.64 | 0.73 | 0.35 | 0.40 | 1.12 | 1.03 | 0.80 |  |
| Microbacteriaceae | 0.81 | 1.91 | 0.49 | 0.28 | 0.09 | 0.14 | 0.31 |  |
| Nocardiaceae | 0.20 | 0.38 | 0.61 | 1.02 | 0.06 | 0.04 | 0.42 |  |
| Nocardioidaceae | 1.18 | 0.22 | 0.02 | 0.02 | 0.07 | 0.04 | 0.01 |  |
| Opitutaceae | 3.88 | 0.04 | 0.00 | 0.01 | 0.18 | 0.18 | 0.12 |  |
| Other 27 - Order: AKYG1722 | 0.01 | 1.04 | 1.24 | 1.57 | 1.75 | 0.96 | 2.00 |  |
| Other 28 - Order: JG30_KF_CM45 | 0.06 | 0.03 | 0.00 | 0.00 | 0.49 | 0.93 | 1.04 |  |
| Other 39 - Order: AT425_EubC11_terrestrial_group | 0.00 | 0.84 | 0.56 | 1.15 | 0.00 | 0.00 | 0.00 |  |
| Other 41 - Class: Gemmatimonadetes | 0.03 | 0.01 | 0.00 | 0.02 | 4.96 | 2.17 | 0.67 |  |
| Other 44 - Phylum: Other | 1.48 | 2.84 | 4.67 | 1.25 | 2.73 | 0.96 | 1.56 |  |
| Other 55 - Order: Rhizobiales | 1.54 | 0.06 | 0.08 | 0.11 | 0.13 | 0.11 | 0.12 |  |
| Other 59 - Order: Burkholderiales | 0.66 | 2.43 | 0.69 | 0.78 | 8.15 | 13.58 | 29.65 |  |
| Other 62 - Class: Betaproteobacteria | 1.23 | 0.01 | 0.00 | 0.00 | 2.06 | 1.26 | 0.92 |  |
| Other 75 - Class: Betaproteobacteria | 1.31 | 0.06 | 0.00 | 0.01 | 0.00 | 0.00 | 0.00 |  |
| Paenibacillaceae | 0.40 | 8.78 | 11.62 | 8.22 | 8.90 | 10.55 | 10.40 |  |
| Phyllobacteriaceae | 0.24 | 2.65 | 0.23 | 0.35 | 0.94 | 1.10 | 1.80 |  |
| Planctomycetaceae | 2.90 | 0.21 | 0.64 | 0.34 | 0.24 | 0.11 | 0.72 |  |
| Planococcaceae | 0.14 | 28.85 | 34.26 | 48.42 | 0.01 | 0.00 | 0.01 |  |
| Rhodobacteraceae | 1.74 | 0.02 | 0.00 | 0.00 | 0.04 | 0.04 | 0.06 |  |
| Rhodospirillaceae | 1.19 | 0.05 | 0.03 | 0.15 | 0.02 | 0.01 | 0.00 |  |
| Sphingobacteriaceae | 1.13 | 0.01 | 0.01 | 0.02 | 1.06 | 0.72 | 1.37 |  |
| Sphingomonadaceae | 0.73 | 1.71 | 0.30 | 0.79 | 1.51 | 1.39 | 6.91 |  |
| Spirochaetaceae | 1.07 | 0.00 | 0.00 | 0.00 | 0.00 | 0.00 | 0.00 |  |
| uncultured_3830 | 1.22 | 0.00 | 0.00 | 0.00 | 0.00 | 0.00 | 0.00 |  |
| Unknown_Family_1002141 | 1.07 | 0.03 | 0.00 | 0.03 | 0.00 | 0.00 | 0.00 |  |
| Unknown_Family_1002345 | 0.00 | 1.21 | 0.45 | 0.57 | 0.00 | 0.00 | 0.00 |  |
| Unknown_Family_1002687 | 1.08 | 0.00 | 0.00 | 0.00 | 0.00 | 0.00 | 0.00 |  |
| Unknown_Family_365 | 3.57 | 0.01 | 0.00 | 0.00 | 0.00 | 0.00 | 0.00 |  |
| Vulgatibacteraceae | 0.00 | 2.50 | 1.54 | 1.08 | 0.34 | 0.33 | 0.18 |  |
| Xanthomonadaceae | 5.03 | 0.16 | 0.01 | 0.01 | 0.97 | 1.37 | 2.94 |  |
| Xanthomonadales_Incertae_Sedis_3825 | 2.85 | 0.73 | 0.14 | 0.60 | 0.09 | 0.12 | 0.13 |  |
| **BE 37°C** | | | | | | | | |
| **Passage** | | | | | | | | |
| **Family** | **0** | **1** | **2** | **3** | **4** | **5** | **6** |  |
| Bacillaceae | 6.15 | 33.98 | 38.17 | 27.88 | 56.78 | 56.80 | 28.63 |  |
| Beijerinckiaceae | 0.00 | 0.31 | 0.10 | 0.08 | 3.58 | 0.50 | 1.36 |  |
| BIrii41 | 3.24 | 0.00 | 0.00 | 0.00 | 0.00 | 0.00 | 0.00 |  |
| Bradyrhizobiaceae | 0.33 | 1.30 | 0.22 | 0.14 | 0.07 | 0.09 | 0.15 |  |
| Brucellaceae | 0.04 | 0.53 | 0.11 | 0.14 | 0.89 | 1.95 | 2.84 |  |
| Caulobacteraceae | 1.24 | 0.62 | 0.03 | 0.01 | 0.04 | 0.11 | 0.29 |  |
| Cellvibrionaceae | 2.28 | 0.00 | 0.00 | 0.00 | 0.00 | 0.00 | 0.00 |  |
| Chitinophagaceae | 3.81 | 0.10 | 0.03 | 0.03 | 0.00 | 0.00 | 0.01 |  |
| Comamonadaceae | 2.50 | 0.19 | 0.02 | 0.02 | 0.14 | 0.05 | 0.05 |  |
| Cytophagaceae | 6.71 | 0.02 | 0.00 | 0.00 | 0.00 | 0.00 | 0.00 |  |
| Fibrobacteraceae | 2.68 | 0.00 | 0.00 | 0.00 | 0.00 | 0.00 | 0.00 |  |
| Flavobacteriaceae | 1.32 | 0.00 | 0.00 | 0.00 | 0.00 | 0.00 | 0.00 |  |
| Hyphomicrobiaceae | 1.64 | 0.73 | 0.35 | 0.40 | 1.12 | 1.03 | 0.80 |  |
| Microbacteriaceae | 0.81 | 1.91 | 0.49 | 0.28 | 0.09 | 0.14 | 0.31 |  |
| Nocardiaceae | 0.20 | 0.38 | 0.61 | 1.02 | 0.06 | 0.04 | 0.42 |  |
| Nocardioidaceae | 1.18 | 0.22 | 0.02 | 0.02 | 0.07 | 0.04 | 0.01 |  |
| Opitutaceae | 3.88 | 0.04 | 0.00 | 0.01 | 0.18 | 0.18 | 0.12 |  |
| Other 27 - Order: AKYG1722 | 0.01 | 1.04 | 1.24 | 1.57 | 1.75 | 0.96 | 2.00 |  |
| Other 28 - Order: JG30_KF_CM45 | 0.06 | 0.03 | 0.00 | 0.00 | 0.49 | 0.93 | 1.04 |  |
| Other 39 - Order: AT425_EubC11_terrestrial_group | 0.00 | 0.84 | 0.56 | 1.15 | 0.00 | 0.00 | 0.00 |  |
| Other 41 - Class: Gemmatimonadetes | 0.03 | 0.01 | 0.00 | 0.02 | 4.96 | 2.17 | 0.67 |  |
| Other 44 - Phylum: Other | 1.48 | 2.84 | 4.67 | 1.25 | 2.73 | 0.96 | 1.56 |  |
| Other 55 - Order: Rhizobiales | 1.54 | 0.06 | 0.08 | 0.11 | 0.13 | 0.11 | 0.12 |  |
| Other 59 - Burkholderiales | 0.66 | 2.43 | 0.69 | 0.78 | 8.15 | 13.58 | 29.65 |  |
| Other 62 - Class: Betaproteobacteria | 1.23 | 0.01 | 0.00 | 0.00 | 2.06 | 1.26 | 0.92 |  |
| Other 75 - Class: Gammaproteobacteria | 1.31 | 0.06 | 0.00 | 0.01 | 0.00 | 0.00 | 0.00 |  |
| Paenibacillaceae | 0.40 | 8.78 | 11.62 | 8.22 | 8.90 | 10.55 | 10.40 |  |
| Phyllobacteriaceae | 0.24 | 2.65 | 0.23 | 0.35 | 0.94 | 1.10 | 1.80 |  |
| Planctomycetaceae | 2.90 | 0.21 | 0.64 | 0.34 | 0.24 | 0.11 | 0.72 |  |
| Planococcaceae | 0.14 | 28.85 | 34.26 | 48.42 | 0.01 | 0.00 | 0.01 |  |
| Rhodobacteraceae | 1.74 | 0.02 | 0.00 | 0.00 | 0.04 | 0.04 | 0.06 |  |
| Rhodospirillaceae | 1.19 | 0.05 | 0.03 | 0.15 | 0.02 | 0.01 | 0.00 |  |
| Sphingobacteriaceae | 1.13 | 0.01 | 0.01 | 0.02 | 1.06 | 0.72 | 1.37 |  |
| Sphingomonadaceae | 0.73 | 1.71 | 0.30 | 0.79 | 1.51 | 1.39 | 6.91 |  |
| Spirochaetaceae | 1.07 | 0.00 | 0.00 | 0.00 | 0.00 | 0.00 | 0.00 |  |
| uncultured_3830 | 1.22 | 0.00 | 0.00 | 0.00 | 0.00 | 0.00 | 0.00 |  |
| Unknown_Family_1002141 | 1.07 | 0.03 | 0.00 | 0.03 | 0.00 | 0.00 | 0.00 |  |
| Unknown_Family_1002345 | 0.00 | 1.21 | 0.45 | 0.57 | 0.00 | 0.00 | 0.00 |  |
| Unknown_Family_1002687 | 1.08 | 0.00 | 0.00 | 0.00 | 0.00 | 0.00 | 0.00 |  |
| Unknown_Family_365 | 3.57 | 0.01 | 0.00 | 0.00 | 0.00 | 0.00 | 0.00 |  |
| Vulgatibacteraceae | 0.00 | 2.50 | 1.54 | 1.08 | 0.34 | 0.33 | 0.18 |  |
| Xanthomonadaceae | 5.03 | 0.16 | 0.01 | 0.01 | 0.97 | 1.37 | 2.94 |  |
| Xanthomonadales_Incertae_Sedis_3825 | 2.85 | 0.73 | 0.14 | 0.60 | 0.09 | 0.12 | 0.13 |  |
| **KRAFT 30°C** | | | | | | | | |
| **Passage** | | | | | | | | |
| **Family** | **0** | **1** | **2** | **3** | **4** | **5** | **6** |  |
| Bacillaceae | 6.15 | 1.43 | 0.07 | 19.62 | 0.03 | 0.01 | 0.00 |  |
| Beijerinckiaceae | 0.00 | 0.92 | 0.89 | 0.74 | 2.03 | 0.54 | 2.04 |  |
| BIrii41 | 3.24 | 0.03 | 0.00 | 0.00 | 0.00 | 0.00 | 0.00 |  |
| Bradyrhizobiaceae | 0.33 | 0.77 | 3.78 | 0.19 | 2.24 | 1.88 | 2.34 |  |
| Burkholderiaceae | 0.04 | 1.40 | 2.28 | 0.15 | 6.07 | 5.14 | 3.43 |  |
| Caulobacteraceae | 1.24 | 2.30 | 1.42 | 0.35 | 0.89 | 0.73 | 1.46 |  |
| Cellvibrionaceae | 2.28 | 0.00 | 0.00 | 0.00 | 0.00 | 0.00 | 0.00 |  |
| Chitinophagaceae | 3.81 | 3.06 | 2.75 | 1.25 | 1.12 | 2.47 | 2.18 |  |
| Comamonadaceae | 2.50 | 1.29 | 0.53 | 0.09 | 0.10 | 0.06 | 0.10 |  |
| Cytophagaceae | 6.71 | 0.02 | 0.02 | 0.00 | 0.03 | 0.02 | 0.02 |  |
| Erythrobacteraceae | 0.43 | 8.17 | 2.88 | 0.50 | 1.53 | 1.57 | 2.42 |  |
| Fibrobacteraceae | 2.68 | 0.00 | 0.00 | 0.00 | 0.00 | 0.00 | 0.00 |  |
| Flavobacteriaceae | 1.32 | 0.00 | 0.00 | 0.00 | 0.07 | 0.01 | 0.00 |  |
| Hyphomicrobiaceae | 1.64 | 1.46 | 10.60 | 0.95 | 6.08 | 5.57 | 6.47 |  |
| Methylobacteriaceae | 0.17 | 2.94 | 13.26 | 1.80 | 27.83 | 27.42 | 22.52 |  |
| Microbacteriaceae | 0.81 | 3.11 | 8.75 | 0.46 | 3.36 | 2.58 | 2.92 |  |
| Nocardioidaceae | 1.18 | 0.65 | 0.51 | 0.09 | 0.25 | 0.18 | 0.16 |  |
| Opitutaceae | 3.88 | 0.17 | 0.39 | 0.07 | 0.34 | 0.26 | 0.42 |  |
| Other 15 - Phylum: Armatimonadetes | 0.00 | 1.18 | 0.19 | 0.00 | 0.00 | 0.00 | 0.00 |  |
| Other 44 - Phylum: Other | 1.48 | 0.12 | 0.47 | 0.51 | 0.25 | 0.40 | 0.24 |  |
| Other 49 - Order: Planctomycetales | 0.54 | 1.67 | 0.59 | 0.90 | 2.03 | 4.50 | 3.21 |  |
| Other 55 - Order: Rhizobiales | 1.54 | 0.83 | 10.64 | 0.70 | 4.11 | 2.17 | 4.36 |  |
| Other 59 - Order: Burkholderiales | 0.66 | 14.14 | 7.56 | 4.35 | 9.04 | 8.32 | 9.76 |  |
| Other 62 - Class: Betaproteobacteria | 1.23 | 0.47 | 0.00 | 0.01 | 0.00 | 0.00 | 0.01 |  |
| Other 75 - Class: Gammaproteobacteria | 1.31 | 0.03 | 0.00 | 0.08 | 0.05 | 0.09 | 0.07 |  |
| Other 85 - Order: Chthoniobacterales | 0.21 | 0.15 | 1.63 | 0.09 | 0.72 | 0.71 | 0.02 |  |
| Paenibacillaceae | 0.40 | 0.81 | 0.39 | 4.60 | 3.27 | 2.58 | 0.40 |  |
| Phyllobacteriaceae | 0.24 | 0.61 | 0.95 | 0.42 | 2.04 | 1.12 | 1.29 |  |
| Planctomycetaceae | 2.90 | 0.51 | 0.03 | 2.09 | 0.00 | 0.00 | 0.00 |  |
| Planococcaceae | 0.14 | 0.06 | 0.02 | 40.05 | 0.00 | 0.00 | 0.00 |  |
| Promicromonosporaceae | 0.42 | 1.42 | 0.91 | 0.05 | 0.52 | 0.07 | 0.10 |  |
| Pseudomonadaceae | 0.24 | 4.07 | 6.83 | 9.31 | 0.96 | 7.30 | 4.49 |  |
| Rhizobiaceae | 0.05 | 0.15 | 1.11 | 0.16 | 5.59 | 8.45 | 3.56 |  |
| Rhodobacteraceae | 1.74 | 0.50 | 0.67 | 0.32 | 0.69 | 0.25 | 0.14 |  |
| Sphingobacteriaceae | 1.13 | 0.46 | 1.36 | 0.44 | 1.61 | 1.88 | 1.45 |  |
| Sphingomonadaceae | 0.73 | 28.58 | 7.05 | 0.70 | 7.03 | 5.84 | 11.56 |  |
| Spirochaetaceae | 1.07 | 0.00 | 0.00 | 0.00 | 0.00 | 0.00 | 0.00 |  |
| uncultured_3830 | 1.22 | 0.00 | 0.00 | 0.47 | 0.00 | 0.00 | 0.00 |  |
| Unknown_Family_1002141 | 1.07 | 0.27 | 0.00 | 0.01 | 0.00 | 0.00 | 0.00 |  |
| Unknown_Family_1002687 | 1.08 | 0.02 | 0.00 | 0.00 | 0.00 | 0.00 | 0.00 |  |
| Unknown_Family_365 | 3.57 | 0.02 | 0.00 | 0.00 | 0.00 | 0.00 | 0.00 |  |
| Vulgatibacteraceae | 0.00 | 0.33 | 1.82 | 0.21 | 0.75 | 0.56 | 0.58 |  |
| Xanthobacteraceae | 0.14 | 0.09 | 1.01 | 0.08 | 0.38 | 0.25 | 0.43 |  |
| Xanthomonadaceae | 5.03 | 5.42 | 1.56 | 0.67 | 1.08 | 0.76 | 2.92 |  |
| Xanthomonadales_Incertae_Sedis_3825 | 2.85 | 1.32 | 0.53 | 0.19 | 0.85 | 0.73 | 0.85 |  |
| **KRAFT 37°C** | | | | | | | | |
| **Passage** | | | | | | | | |
| **Family** | **0** | **1** | **2** | **3** | **4** | **5** | **6** |  |
| Bacillaceae | 6.15 | 1.02 | 0.04 | 0.01 | 0.00 | 0.00 | 0.00 |  |
| Beijerinckiaceae | 0.00 | 0.20 | 0.99 | 0.70 | 1.32 | 0.97 | 1.30 |  |
| BIrii41 | 3.24 | 0.00 | 0.00 | 0.00 | 0.00 | 0.00 | 0.00 |  |
| Bradyrhizobiaceae | 0.33 | 1.70 | 6.65 | 6.82 | 4.38 | 2.01 | 1.70 |  |
| Brucellaceae | 0.04 | 5.26 | 7.87 | 5.45 | 1.87 | 2.21 | 2.54 |  |
| Burkholderiaceae | 0.04 | 2.23 | 8.82 | 13.24 | 11.07 | 4.93 | 3.90 |  |
| Caulobacteraceae | 1.24 | 0.14 | 0.10 | 0.13 | 0.14 | 0.12 | 0.09 |  |
| Cellvibrionaceae | 2.28 | 0.00 | 0.00 | 0.00 | 0.00 | 0.00 | 0.00 |  |
| Chitinophagaceae | 3.81 | 1.58 | 0.00 | 0.00 | 0.00 | 0.00 | 0.00 |  |
| Comamonadaceae | 2.50 | 0.09 | 0.02 | 0.00 | 0.00 | 0.00 | 0.00 |  |
| Cytophagaceae | 6.71 | 0.05 | 0.00 | 0.00 | 0.00 | 0.00 | 0.00 |  |
| Enterobacteriaceae | 0.04 | 9.32 | 0.26 | 0.00 | 0.00 | 0.00 | 0.00 |  |
| Erythrobacteraceae | 0.43 | 16.11 | 5.05 | 4.48 | 4.49 | 2.81 | 2.67 |  |
| Fibrobacteraceae | 2.68 | 0.00 | 0.00 | 0.00 | 0.00 | 0.00 | 0.00 |  |
| Flavobacteriaceae | 1.32 | 0.00 | 0.00 | 0.00 | 0.00 | 0.00 | 0.00 |  |
| Hyphomicrobiaceae | 1.64 | 1.10 | 4.23 | 4.22 | 4.28 | 4.47 | 5.82 |  |
| Methylobacteriaceae | 0.17 | 0.87 | 1.15 | 1.41 | 1.09 | 0.77 | 0.79 |  |
| Microbacteriaceae | 0.81 | 1.70 | 2.27 | 2.74 | 1.19 | 1.62 | 1.99 |  |
| Mycobacteriaceae | 0.32 | 0.68 | 13.24 | 13.56 | 9.26 | 6.82 | 8.47 |  |
| Nocardiaceae | 0.20 | 0.20 | 9.50 | 5.74 | 10.95 | 6.46 | 7.83 |  |
| Nocardioidaceae | 1.18 | 1.13 | 0.09 | 0.11 | 0.03 | 0.05 | 0.04 |  |
| Opitutaceae | 3.88 | 0.58 | 1.63 | 0.81 | 0.50 | 0.81 | 0.90 |  |
| Other 44 - Phylum: Other | 1.48 | 0.17 | 0.06 | 0.05 | 0.02 | 0.02 | 0.02 |  |
| Other 55 - Order: Rhizobiales | 1.54 | 0.57 | 2.31 | 2.55 | 1.94 | 1.75 | 1.83 |  |
| Other 59 - Order: Burkholderiales | 0.66 | 14.93 | 5.96 | 3.88 | 3.51 | 2.58 | 2.31 |  |
| Other 62 - Class: Betaproteobacteria | 1.23 | 5.21 | 0.71 | 0.88 | 0.73 | 0.36 | 0.17 |  |
| Other 75 - Class: Gammaproteobacteria | 1.31 | 0.00 | 0.00 | 0.00 | 0.00 | 0.00 | 0.00 |  |
| Phyllobacteriaceae | 0.24 | 2.53 | 4.16 | 7.65 | 26.11 | 44.39 | 44.36 |  |
| Planctomycetaceae | 2.90 | 0.24 | 0.00 | 0.00 | 0.00 | 0.00 | 0.00 |  |
| Promicromonosporaceae | 0.42 | 1.84 | 6.40 | 5.19 | 3.43 | 3.32 | 4.29 |  |
| Pseudomonadaceae | 0.24 | 4.21 | 0.12 | 0.03 | 0.04 | 0.00 | 0.01 |  |
| Rhodobacteraceae | 1.74 | 0.03 | 0.00 | 0.00 | 0.00 | 0.00 | 0.00 |  |
| Rhodospirillaceae | 1.19 | 1.28 | 1.02 | 0.64 | 1.03 | 0.85 | 0.55 |  |
| Sphingobacteriaceae | 1.13 | 0.00 | 0.00 | 0.00 | 0.00 | 0.00 | 0.00 |  |
| Sphingomonadaceae | 0.73 | 8.13 | 8.12 | 11.70 | 6.64 | 8.19 | 4.97 |  |
| Spirochaetaceae | 1.07 | 0.00 | 0.00 | 0.00 | 0.00 | 0.00 | 0.00 |  |
| uncultured_3830 | 1.22 | 0.00 | 0.00 | 0.00 | 0.00 | 0.00 | 0.00 |  |
| Unknown_Family_1002141 | 1.07 | 0.00 | 0.00 | 0.00 | 0.00 | 0.00 | 0.00 |  |
| Unknown_Family_1002687 | 1.08 | 0.00 | 0.00 | 0.00 | 0.00 | 0.00 | 0.00 |  |
| Unknown_Family_365 | 3.57 | 0.00 | 0.00 | 0.00 | 0.00 | 0.00 | 0.00 |  |
| Xanthomonadaceae | 5.03 | 11.88 | 5.54 | 1.82 | 3.52 | 2.72 | 1.75 |  |
| Xanthomonadales_Incertae_Sedis_3825 | 2.85 | 0.47 | 0.27 | 0.11 | 0.10 | 0.05 | 0.04 |  |
| Acetobacteraceae | 0.11 | 0.12 | 0.46 | 3.25 | 0.11 | 0.08 | 0.04 |  |
