# Supplementary material for: Bacterial diversity dynamics in microbial consortia selected for lignin utilization
Source: PLoS One. 2021 Sep 13;16(9):e0255083. doi: 10.1371/journal.pone.0255083 (PMC8437272; doi:10.1371/journal.pone.0255083)
Supplement: S1 File — Bray Curtis matrix PERMANOVA analysis was performed using the adonis function of vegan package in R. (DOCX) [file pone.0255083.s006.docx]

**S1 file**. Variance of the Bray Curtis dissimilarities matrix associated with substrate (base extracted and Kraft lignin), temperature (30 °C and 37 °C), Passage (0 to 6) and soil (MG and BY). Bray Curtis matrix PERMANOVA analysis was performed using the adonis function of vegan package in R.

**SUBSTRATE (lignin type)**

Call:

adonis(formula = dist.bray ~ meta_all$Substrate, permutations = 10000)

Permutation: free

Number of permutations: 10000

Terms added sequentially (first to last)

Df SumsOfSqs MeanSqs F.Model R2 Pr(>F)

meta_all$Substrate 2 4.4027 2.20135 11.345 0.32559 9.999e-05 ***

Residuals 47 9.1193 0.19403 0.67441

Total 49 13.5220 1.00000

---

Signif. codes: 0 ‘***’ 0.001 ‘**’ 0.01 ‘*’ 0.05 ‘.’ 0.1 ‘ ’ 1

**TEMPERATURE**

Call:

adonis(formula = dist.bray ~ meta_all$Temperature, permutations = 10000)

Permutation: free

Number of permutations: 10000

Terms added sequentially (first to last)

Df SumsOfSqs MeanSqs F.Model R2 Pr(>F)

meta_all$Temperature 2 1.6559 0.82795 3.2794 0.12246 0.0006999 ***

Residuals 47 11.8661 0.25247 0.87754

Total 49 13.5220 1.00000

---

Signif. codes: 0 ‘***’ 0.001 ‘**’ 0.01 ‘*’ 0.05 ‘.’ 0.1 ‘ ’ 1

**PASSAGE**

Call:

adonis(formula = dist.bray ~ meta_all$Passage, permutations = 10000)

Permutation: free

Number of permutations: 10000

Terms added sequentially (first to last)

Df SumsOfSqs MeanSqs F.Model R2 Pr(>F)

meta_all$Passage 6 1.2053 0.20089 0.70134 0.08914 0.9197

Residuals 43 12.3167 0.28643 0.91086

Total 49 13.5220 1.00000

**SOIL**

Call:

adonis(formula = dist.bray ~ meta_all$Soil, permutations = 10000)

Permutation: free

Number of permutations: 10000

Terms added sequentially (first to last)

Df SumsOfSqs MeanSqs F.Model R2 Pr(>F)

meta_all$Soil 1 0.6916 0.69156 2.5872 0.05114 0.0246 *

Residuals 48 12.8305 0.26730 0.94886

Total 49 13.5220 1.00000

---

Signif. codes: 0 ‘***’ 0.001 ‘**’ 0.01 ‘*’ 0.05 ‘.’ 0.1 ‘ ’ 1

SUBSTRATE*TEMPERATURE

Call:

adonis(formula = dist.bray ~ meta_all$Temperature * meta_all$Description, permutations = 10000)

Permutation: free

Number of permutations: 10000

Terms added sequentially (first to last)

Df SumsOfSqs MeanSqs F.Model R2 Pr(>F)

meta_all$Temperature 2 1.6559 0.8280 5.0276 0.12246 9.999e-05 ***

meta_all$Description 1 3.7881 3.7881 23.0030 0.28015 9.999e-05 ***

meta_all$Temperature:meta_all$Description 1 0.6674 0.6674 4.0527 0.04936 0.0034 **

Residuals 45 7.4106 0.1647 0.54804

Total 49 13.5220 1.00000

---

Signif. codes: 0 ‘***’ 0.001 ‘**’ 0.01 ‘*’ 0.05 ‘.’ 0.1 ‘ ’ 1

**ALL VARIABLES**

Call:

Adonis (formula = dist.bray ~ meta_all$Temperature * meta_all$Soil * meta_all$Passage * meta_all$Substrate, permutations = 10000)

Permutation: free

Number of permutations: 10000

Terms added sequentially (first to last)

|  | Df | SumsOfSqs | MeanSqs | F.Model | R2 | Pr(>F) |
| --- | --- | --- | --- | --- | --- | --- |
| meta_all$Temperature | 2 | 1.6559 | 1 | 0 | 0.12246 | 1 |
| meta_all$Soil | 1 | 0.6916 | 1 | 0 | 0.05114 | 1 |
| meta_all$Passage | 5 | 0.5908 | 0 | 0 | 0.04369 | 1 |
| meta_all$Substrate | 1 | 3.7881 | 4 | 0 | 0.28015 | 1 |
| meta_all$Temperature:meta_all$Soil | 2 | 0.9504 | 0 | 0 | 0.07028 | 1 |
| meta_all$Temperature:meta_all$Passage | 5 | 0.4060 | 0 | 0 | 0.03002 | 1 |
| meta_all$Soil:meta_all$Passage | 5 | 0.6391 | 0 | 0 | 0.04727 | 1 |
| meta_all$Temperature:meta_all$Substrate | 1 | 0.6674 | 1 | 0 | 0.04936 | 1 |
| meta_all$Soil:meta_all$Substrate | 1 | 1.0216 | 1 | 0 | 0.07555 | 1 |
| meta_all$Passage:meta_all$Substrate | 5 | 0.7275 | 0 | 0 | 0.05380 | 1 |
| meta_all$Temperature:meta_all$Soil:meta_all$Passage | 5 | 0.3110 | 0 | 0 | 0.02300 | 1 |
| meta_all$Temperature:meta_all$Soil:meta_all$Substrate | 1 | 0.2808 | 0 | 0 | 0.02077 | 1 |
| meta_all$Temperature:meta_all$Passage:meta_all$Substrate | 5 | 0.6496 | 0 | 0 | 0.04804 | 1 |
| meta_all$Soil:meta_all$Passage:meta_all$Substrate | 5 | 0.7776 | 0 | 0 | 0.05751 | 1 |
| meta_all$Temperature:meta_all$Soil:meta_all$Passage:meta_all$Substrate | 5 | 0.3648 | 0 | 0 | 0.02698 | 1 |
| Residuals | 0 | 0.0000 | -Inf | 0.00000 |  |  |
| Total | 49 | 13.5220 |  | 1.00000 |  |  |
