# Supplementary material for: Bacterial diversity dynamics in microbial consortia selected for lignin utilization
Source: PLoS One. 2021 Sep 13;16(9):e0255083. doi: 10.1371/journal.pone.0255083 (PMC8437272; doi:10.1371/journal.pone.0255083)
Supplement: S1 Data — (DOCX) [file pone.0255083.s008.docx]

**Fig 4. Venn diagram of bacterial genera present at the 6^th^ passage of enrichment in consortia obtained using either base-extracted (BE-Lig) or Kraft lignin (Kraft) as carbon source at cultivation temperatures of 30 °C and 37 °C.** Shown in parenthesis is the number of genera for each growth condition with relative abundance above 1%. (A) Bacterial consortia derived from MG compost. (B) Bacterial consortia derived from backyard (BY) compost.
